# Rbfox3 Promotes Transformation of MDSC‐Like Tumor Cells to Shape Immunosuppressive Microenvironment

**DOI:** 10.1002/advs.202404585

**Published:** 2025-01-07

**Authors:** Zhiyang Li, Zhuangzhuang Feng, Mengzhan Chen, Xinxiu Shi, Bijia Cui, Yujie Sun, Heng Zhang, Yinan Li, Caihong Chen, Yiqian Feng, Jingxia Han, Xuewu Xing, Huijuan Liu, Tao Sun

**Affiliations:** ^1^ State Key Laboratory of Medicinal Chemical Biology and College of Pharmacy Nankai University Tianjin 300450 China; ^2^ Tianjin Key Laboratory of Early Druggability Evaluation of Innovative Drugs Tianjin International Joint Academy of Biomedicine Tianjin 300457 China; ^3^ Department of Orthopedics Tianjin First Central Hospital Tianjin 300190 China

**Keywords:** immunosuppressive tumor microenvironment, MDSC‐like tumor cells, phase‐separated particles, Rbfox3

## Abstract

Myeloid‐derived suppressor cells (MDSCs) within the tumor microenvironment (TME) contribute to the malignant progression of tumors by exerting immunosuppressive effects. Bacterial lipopolysaccharides (LPS) have been widely demonstrated in various types of solid tumors. LPS can promote the malignant progression of tumors, which mechanism has not yet been fully elucidated. In this study, a type of MDSC‐like tumor cells (MLTCs) is found in tumor tissues induced by low‐dose and long‐term LPS stimulation. MLTCs can simultaneously express tumor cell and MDSCs markers. Similar to MDSCs, MLTCs can produce arginine, nitric oxide, and reactive oxygen species and inhibit the activity of NK and T cells to promote the formation of an immunosuppressive microenvironment. MLTCs can also promote tumor cell proliferation and vasculogenic mimicry formation. CRISPR‐Cas9 activity screening studies identified RNA‐binding Fox‐1 homolog 3 (Rbfox3) as a critical protein for MLTCs formation after LPS treatment. Rbfox3 can transcriptionally regulate the expression of Ass1 in the form of phase‐separated particles. Crocin can inhibit the generation of MLTCs by disrupting phase‐separated particles of Rbfox3 and enhance the anti‐tumor effects of immune checkpoint inhibitors (ICIs).

## Graphical abstract

The Long‐term low‐dose injection of LPS in tumor‐bearing mice caused Rbfox3 phase separation, resulting in the transformation of tumor cells into MDSC‐like tumor cells (MLTCs), possess potent immunosuppressive capabilities and promote tumor cell proliferation, migration, and invasion, thereby facilitating the malignant progression of the tumor.

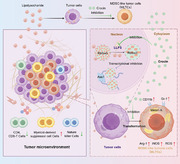

## 1 Introduction

Myeloid‐derived suppressor cells (MDSCs) are heterogeneous cells originating from bone marrow.^[^ ^1^ ^]^ In the tumor microenvironment (TME), a substantial increase in the number of MDSCs promotes tumor growth.^[^ ^2^, ^3^ ^]^ In adaptive immunity, MDSCs competitively deplete cysteine in the in vivo environment and upregulate the metabolic activity of inducible nitric oxide synthase (iNOS) and Arg‐1, thereby depleting l‐arginine, blocking T cell generation, and suppressing T cell immune responses by producing reactive oxygen species (ROS).^[^ ^4^, ^5^ ^]^ MDSCs reduce the number of natural killer (NK) cells and inhibit NK cell function.^[^ ^6^, ^7^, ^8^ ^]^ Furthermore, MDSCs directly utilize Vascular Endothelial Growth Factor (VEGF) in the TME to promote tumor growth, development, and vasculogenic mimicry. MDSCs also secrete matrix metalloproteinase (e.g. MMP2 and MMP9), which induce epithelial–mesenchymal transition (EMT), migration, and invasion of tumor cells.^[^ ^9^, ^10^ ^]^

Granulocytic/polymorphonuclear and monocytic MDSCs are classified according to their origin in the granulocytic or monocytic lineage.^[^ ^11^ ^]^ Single‐cell sequencing has shown that different subtypes of MDSCs have different functions, with polymorphonuclear myeloid‐derived suppressor cells (PMN‐MDSCs) being most specific to tumor sites.^[^ ^6^ ^]^ However, the complexity of MDSCs limits the insight into the function and origin of this cell population.

Cancer cells have properties similar to those of embryonic cells and exhibit some plasticity.^[^ ^12^ ^]^ Cancer cells can express stem cell genes, including nodal, Oct4, Sox2, and Nanog, to generate cancer stem‐like cells.^[^ ^13^ ^]^ A classic example that supports cancer cell differentiation is the differentiation of breast cancer cells into adipocytes upon long‐term induction.^[^ ^14^, ^15^ ^]^ Melanomas mainly originate from melanocytes, and melanocytic embryos are derived from neural crest cells with well‐differentiated properties and a strong capacity for differentiation, resulting in a high degree of plasticity.^[^ ^16^, ^17^, ^18^ ^]^ However, whether melanoma cells can transform into MDSC‐like cells has not yet been reported.

LPS has been detected in six common solid tumors, including breast, melanoma, etc.^[^ ^19^ ^]^ LPS can promote tumor growth, migration, and invasion by inducing toll‐like receptor 4 (TLR4) activation,^[^ ^20^ ^]^ inhibit the anti‐tumor function of immune cells, and aggravating the malignant evolution of tumors.^[^ ^21^ ^]^ A survey of patients with pancreatic ductal adenocarcinoma (PDAC) found that serum LPS content was significantly positively correlated with the expression of tumor programmed cell death 1 ligand 1 (PD‐L1). In patients with colorectal cancer (CRC), the concentration of LPS in the blood and tumor tissues was significantly higher than in healthy individuals.^[^ ^22^ ^]^ Additionally, LPS in the chronic hepatitis microenvironment may contribute to the development of liver cancer by modulating the plasticity of liver stem cells (HPCs).^[^ ^23^ ^]^ Therefore, we speculated that LPS may also regulate the plasticity of tumor cells, thereby promoting an increase in MDSCs in the tumor microenvironment and inducing an immunosuppressive microenvironment.

In mice, MDSCs specifically express Gr‐1 and CD11b, which are considered markers defining MDSCs.^[^ ^11^ ^]^ This study showed that chronic inflammatory conditions mimicked by long‐term/low‐dose injection of LPS could induce tumor cells to highly express Gr‐1 and CD11b in B16 tumor‐bearing mice, which were named MDSC‐like tumor cells (MLTCs). In the present study, we characterized MLTCs after LPS treatment and investigated the molecular mechanisms underlying their production. Using the CRISPR‐Cas9 active technology, we found that RNA‐binding Fox‐1 homolog 3 (*Rbfox3*) was a key driver of MLTCs transformation after LPS treatment in B16 cells. After induction with LPS, tumor cells highly express the transcription factor Rbfox3 and transcriptionally repress argininosuccinate synthase 1 (Ass1) expression in a phase‐separated manner, promoting the transformation of tumor cells into MLTCs. MLTCs can inhibit T cell proliferation and NK cell activity, and promote tumor cells growth and vascular mimicry, which can promote malignant progression and immune escape of tumors. To identify drugs that can inhibit MLTCs, crocin was screened by targeting the Rbfox3 protein. Crocin can inhibit the generation of MLTCs and malignant progression of melanoma.

## 2 Result

### 2.1 LPS can Induce B16 Cells to Transform into MDSC‐Like Tumor Cells (MLTCs)

**Figure 1:**
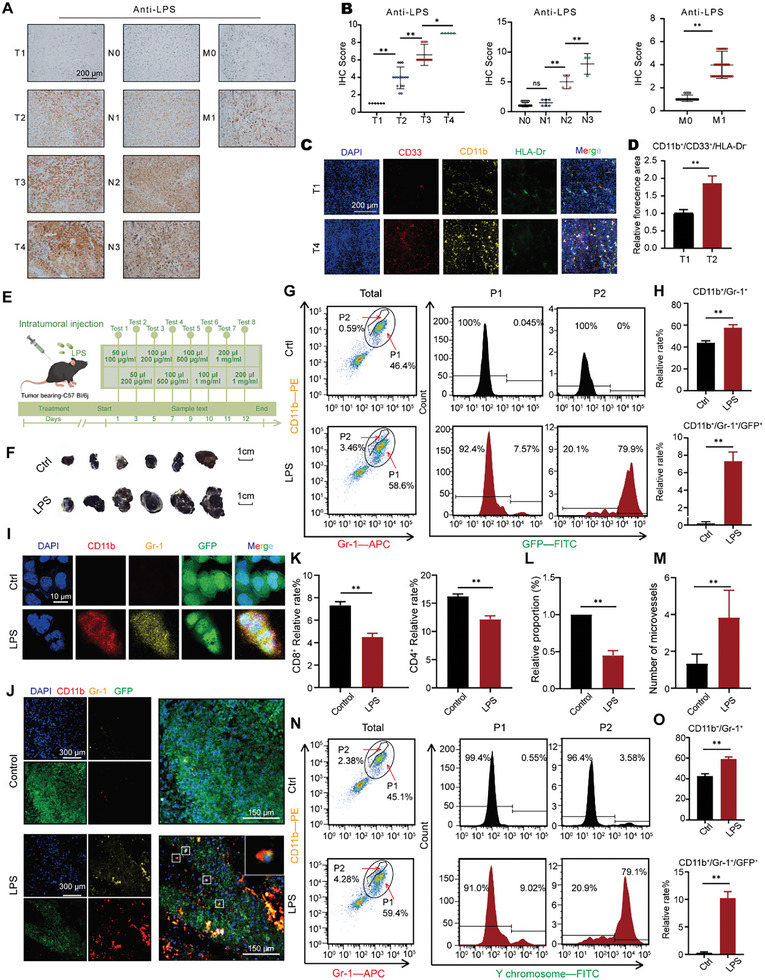
Intratumoral injection of LPS in tumor‐bearing mice promotes the transformation of melanoma tumor cells into MLTCs. A) Immunohistochemical analysis of LPS in melanoma tissues from clinical patients of different pathological stages. B) Quantitative analysis of LPS in melanoma tissues clinical patients. C) Immunofluorescence plots for melanoma tissues clinical sources (CD11b, CD33, HLA‐Dr, and DAPI). D) Fluorescence intensity histogram of MDSCs (CD11b^+^/CD33^+^/HLA‐Dr^−^). E) A schematic of intratumoral LPS injection into tumor‐bearing mice. F) Representative tumor images of tumor‐bearing mice after the intratumoral injection of LPS. G) Flow cytometry was performed to detect CD11b and Gr‐1 signals in tumor cells isolated from tumor‐bearing mice injected with or without LPS intratumorally, and CD11b/Gr‐1 double‐positive cells were gated again to detect GFP signals. H) CD11b, Gr‐1, and GFP‐positive cells were quantified. I) Immunofluorescence plots of tumor cells from B16–EGFP tumor‐bearing mice intratumorally injected with LPS (CD11b, Gr‐1, GFP, and DAPI). J) Trichrome fluorescence micrographs of tumor tissue sections from B16–EGFP tumor‐bearing mice intratumorally injected with LPS. K) Contents of CD8^+^ and CD4^+^ positive cells were presented. L) Relative proportions of the T cell fluorescence of tumor tissue sections were presented. M) Number of microvessels from tumor tissue sections of different groups presented in histogram form. N) After the subcutaneous injection of B16, flow cytometry was performed to detect the CD11b and Gr‐1 signals in the tumors of mice in different groups, and the CD11b/Gr‐1‐double‐positive cells were gated again to detect the FITC signals of Y chromosomal. O) CD11b, Gr‐1, and Y chromosomal positive cells were quantified. All values are presented as the mean ± SD, n = 6, *p < 0.05, **p < 0.01.

The presence of bacterial LPS in different types of solid tumors has been widely demonstrated, and LPS can promote the malignant progression of tumors.^[^ ^19^ ^]^ In addition, some tumor cells have stem cell characteristics that result in high plasticity and promote tumor heterogeneity production.^[^ ^24^ ^]^ As one of the main immunosuppressive cells in the TME, MDSCs production is closely associated with tumor progression.^[^ ^25^ ^]^ Therefore, we investigated the association between tumor cells, LPS stimulation, and MDSCs. We first used immunohistochemistry to analyze the concentration of LPS in clinical tissue samples from patients with melanoma at different pathological stages (**Figure** **1**A). The quantification results revealed that the LPS concentration was positively correlated with the TNM stage (Figure 1B). We used immunofluorescence to assess the abundance of MDSCs in the samples (Figure 1C). The results indicated that the number of MDSCs positively correlated with disease progression (Figure 1D). These findings showed that both LPS and MDSCs levels were positively correlated with the malignant progression of melanoma. To investigate the relationship between LPS and increased MDSCs content, we conducted further studies using a B16‐EGFP tumor‐bearing mice model combined with intratumoral LPS administration (Figure 1E).

Compared with the control group, the tumor volume in the LPS‐treated group exhibited a significant increase, nearly doubling in size. Body weight decreased, and the mouse status score was significantly reduced in the LPS‐treated group compared to the control group (Figure 1F; Figure S1A, Supporting Information). B16‐EGFP tumor tissues were separated into single‐cell suspensions for MDSCs content detection. Flow cytometry analysis demonstrated that compared to the control group, the proportion of MDSCs (CD11b^+^ and Gr‐1^+^) increased from 46.4% to 58.6% in the tumor tissues of the LPS‐treated group (Figure 1G,H). The proportion of splenic MDSCs in tumor‐bearing mice injected with LPS reached 34.4%, which was higher than that in the control group. The proportion of MDSCs in the bone marrow increased from 23.6% to 50% (Figure S1B, Supporting Information). Isolated CD11b/Gr‐1 double‐positive cells were analyzed using flow cytometry to determine their origin. For clarity, cell group P1 represents CD11b/Gr‐1 double‐positive cells (Figure 1G). Surprisingly, ≈8% of all P1 cells in the LPS‐treated group exhibited a GFP signal. However, almost no GFP signal was detected in the control group without LPS induction. In addition, a subset of cells within the P1 population displaying a stronger CD11b signal was designated as the P2 population (Figure 1G). Compared with the control group, the P2 group had a stronger CD11b‐positive signal and a higher proportion of cells with GFP‐positive signals, reaching nearly 80%. To ensure that GFP‐positive cells in P1/P2 cell populations are not due to fluorescence leakage and cross‐staining. We constructed the LPS‐induced B16 tumor‐bearing mice model without a GFP fluorescence signal, CD11b/ Gr‐1 double positive cells were obtained by flow cytometry sorting, and whether CD11b/ Gr‐1 double positive cells contained melanoma antigen A (Melan‐A) specific to B16 cells was detected. Western Blot results showed that Melan‐A was expressed in CD11b/Gr‐1 double‐positive cells in tumor tissues of B16 tumor‐bearing mice induced by LPS (Figure S1B, Supporting Information). The flow cytometry analysis revealed that the CD11b/Gr‐1 double‐positive cell population did not exhibit a green fluorescence signal (Figure S1C, Supporting Information). These results fully demonstrate the presence of GFP‐positive tumor cells in CD11b and Gr‐1 double‐positive cells (MLTCs) in LPS‐induced B16‐EGFP tumor tissues (Figure 1G).

Subsequently, we detected CD11b/Gr‐1 double‐positive cells isolated from the spleen and bone marrow; however, no obvious GFP signal was detected (Figure S1D, Supporting Information). Immunofluorescence experiments further confirmed that some tumor cells in mice treated with LPS had not only their own GFP signals, but also positive CD11b and Gr‐1 signals (Figure 1I). These results showed that LPS treatment may induce CD11b/Gr‐1 double‐positive expression in tumor cells. The results also demonstrated that these cells were limited to the tumor tissue and did not spread to the spleen or bone marrow. For further validation, immunofluorescence analysis was conducted on tumor tissue slices, which demonstrated the colocalization of Gr‐1, CD11b, and GFP signals in a single cell (Figure 1J). Based on these results, we hypothesized that LPS induces the transformation of a subset of B16 cells into CD11b/Gr‐1 double‐positive MDSC‐like cells. The sex chromosome tracing method was used to further verify our findings and avoid false‐positive results due to the phagocytosis of tumor cell surface GFP by MDSCs. Male B16 cells were injected into female C57BL/6 mice, and the tumors were prepared into a single‐cell suspension. Flow cytometry analysis revealed that the proportion of CD11b/Gr‐1 double‐positive cells increased. CD11b/Gr‐1 double‐positive cells in the experimental group presented Y chromosome signals, whereas no relevant signals were detected in the control group (Figure 1N,O). The immunofluorescence results corroborated the flow cytometry data (Figure S1H, Supporting Information). These results confirmed our hypothesis that in the tumor‐bearing mice, LPS can induce the transformation of B16 cells into MDSC‐like tumor cells, which we named MDSC‐like tumor cells (MLTCs). The experimental group showed an increase in tumor volume and a reduction in survival rate. Subsequently, the number of cytotoxic T cells in tumor tissues was detected. Compared with the control group, CD8^+^ and CD4^+^ cells were significantly reduced in the LPS‐treated group (Figure 1K). Immunofluorescence staining of mature T lymphocytes in the tumor tissue sections showed that the fluorescence intensity of the LPS‐treated group was weaker than that of the control group, indicating a lower number of T cells (Figure 1L; Figure S1G, Supporting Information). HE staining of tumor sections demonstrated that the LPS‐treated group had more vessels than the control group (Figure 1M; Figure S1F, Supporting Information).

Next, we attempted to simulate the in vitro microenvironment that induces MLTCs generation. Since MLTCs only occur in the tumor microenvironment, the tumor extract is likely to be a key factor in inducing MLTCs in vitro. By inducing B16 cells with LPS and tumor extract every day, Gr‐1 and CD11b double‐positive cells began to appear after 15 days (Figure S2A,B, Supporting Information). We find that LPS and tumor extract co‐induction can induce about 10% of B16 cells into CD11b/Gr‐1 double‐positive cells. To further investigate MLTCs, all MLTCs were obtained from P1 cells with high GFP expression in the in vivo experiments. We further verified whether MLTCs have a high proliferative capacity similar to that of B16 cells. The MLTCs exhibited decreased activity within 64 h, as assessed by the CCK8 assay (Figure S3A, Supporting Information), and demonstrated a lack of proliferative ability, as confirmed by the clonogenic assay (Figure S3B, Supporting Information).

### 2.2 MLTCs Mimic MDSCs to Execute the Immunosuppressive Function and Promote the Malignant Progression of Tumor Cells In Vitro

**Figure 2:**
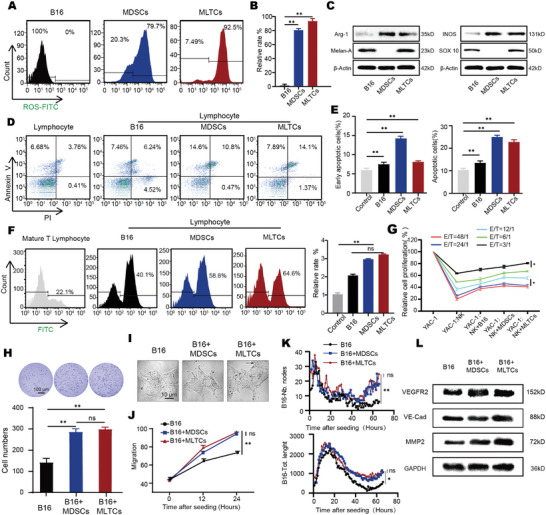
Similar to MDSCs, MLTCs have immunosuppressive capacity and the ability to promote tumor cell proliferation. A–B) ROS levels in B16 cells, MDSCs, and MLTCs groups were measured by flow cytometry and statistical analysis was performed. C) Protein expression levels of iNOS and Arg‐1 in B16 cells, MDSCs, and MLTCs were detected using Western Blot analysis. D, E) B16 cells, MDSCs, and MLTCs co‐cultured with lymphocytes. Annexin‐V/PI staining was used to distinguish early apoptotic cells (Annexin‐V^+^/PI^−^) from late apoptotic cells (Annexin‐V^+^/PI^+^). Quantification of early apoptotic cells (right) versus late apoptotic cells (left) were presented. F) B16 cells, MDSCs, and MLTCs were co‐cultured with CFSE‐labeled mature T lymphocytes, and CFSE dilution was used to measure mature T lymphocyte proliferation. The quantification results of the CFSE‐labeled cells with inhibited proliferative capacity were presented. G) After B16 cells, MDSCs, and MLTCs were co‐cultured with NK cells for 48 h, the effector (NK cells) and target (YAC‐1 cells) cells were divided into five effective target ratios (E/T: 48/1, 24/1, 12/1, 6/1, and 3/1) and co‐cultured for 48 h. The proliferative ability of YAC‐1 cells was detected using the LDH assay. H) Representative plots of cell clonogenicity after coculture MDSCs or MLTCs with B16. I) Representative images of B16 cell morphology: B16 cells, B16 cells co‐cultured with MDSCs, and B16 cells co‐cultured with MLTCs. J) The wound‐healing assay results of cocultured cells is presented in the form of line graphs. K) Quantification of key vascularization measures extreme and Tot. length used to represent the vascularization ability of the co‐cultured cells was presented as line graphs. L) Protein expression levels of VEFGR2, VE‐Cad, MMP2, and MMP9 were detected by Western Blot analysis. All values are presented as the mean ± SD, n = 6, ns = not significant, *p < 0.05, **p < 0.01.

MDSCs inhibit lymphocytes by secreting Arg‐1, iNOS, and ROS. They can also inhibit T cells and NK cells, causing tumor immune escape and immune suppression. MDSCs can also improve the survival of tumor cells and promote vascular mimicry.^[^ ^26^ ^]^ We speculate that MLTCs have functions similar to MDSCs. MLTCs were isolated from the tumor tissues of tumor‐bearing mice treated with LPS, and MDSCs were obtained from the bone marrow of tumor‐bearing mice. The ROS level in MLTCs was significantly higher than that in B16 cells and similar to that in MDSCs (**Figure** **2**A,B). Western Blot analysis indicated that MLTCs had higher Arg‐1 and iNOS expression levels than B16 cells (Figure 2C; Figure S4A, Supporting Information). Therefore, we hypothesized that MLTCs could exert the same effects as MDSCs by expressing Arg‐1, iNOS, or ROS. We also detected the characteristic protein of melanoma, Melan‐A. The results demonstrated that MLTCs expressed characteristic melanocyte proteins (Figure 2C). We speculated that MLTCs not only express related proteins as MDSCs, but also have immunosuppressive activity. Subsequently, we performed an apoptosis assay using Annexin V/PI double staining. Briefly, lymphocytes in the peripheral blood of mice were extracted and co‐cultured with equal amounts of B16 cells, MDSCs, or MLTCs (1:1) under noncontact conditions for 48 h. The results showed that MLTCs promoted lymphocyte apoptosis (Figure 2D,E). The effect of MLTCs on the proliferation of mature lymphocytes was tested using CFSE staining. Mature T lymphocytes were co‐culture with B16 cells, MDSCs, and MLTCs (1:1). The ability of MLTCs to inhibit mature T lymphocyte proliferation was stronger than that of MDSCs or B16 cells (Figure 2F). Mouse NK cells were obtained using an NK cell extraction kit and NK cells were isolated after co‐culture with B16, MDSCs, or MLTCs. Co‐culturing of treated effector cells (NK cells) with target cells (YAC‐1 cells) showed that MLTCs inhibited the cytotoxic activity of NK cells (Figure 2G). These experiments confirm the immunosuppressive function of MLTCs.

MDSCs can stimulate tumor cell growth, development, and vasculogenic mimicry. MLTCs and MDSCs were co‐cultured with normal B16 cells separately. The number of B16 cell clones formed in the MLTC co‐culture group was more than twice that of the control group (Figure 2H). Meanwhile, we found that B16 cells in the co‐culture group had long pseudopodia (Figure 2I), a marker of strong invasive ability.^[^ ^27^ ^]^ In the scratch test, the wound‐healing ability of the experimental group co‐cultured with MLTCs was faster than that of the control group. (Figure 2J; Figure S4B, Supporting Information). We examined the effect of MLTCs on vascular mimicry. Nb.nodes, which represent branching capacity, and Tot.length, which represents tube formation capacity, are recognized as measures of vasculogenic mimicry. B16 cells co‐cultured with MLTCs or MDSCs exhibited stronger vasculogenic mimicry (Figure 2K). Western Blot analysis showed that the co‐cultured with MLTCs or MDSCs groups expressed higher levels of VEGFR2, VE‐cadherin, and MMP2 (Figure 2L; Figure S4C, Supporting Information). The above experiments indicate that MLTCs have immunosuppressive functions and promote tumor cell clonogenesis, migration, and vascular mimicry.

### 2.3 MLTCs Exert Immunosuppressive Effects and Promote Tumor Growth and Metastasis In Vivo

To confirm the ability of MLTCs to promote tumor metastasis in vivo, we established a lung metastasis mouse model and found that the metastatic lesions of the intratumor‐injected MLTCs group were significantly higher than those of the MDSCs group and nearly seven times that of the control group (Figure S5A, Supporting Information). To verify the effect of MLTCs on tumor growth, a B16 tumor‐bearing C57BL/6 mouse model was established. MLTCs, MDSCs, and PBS were administered via intratumoral injection. The tumor volume in the intratumor‐injected MDSCs or MLTCs group was significantly larger than that in the control group, and the body weight of the mice decreased by 20% (Figure S5B,C, Supporting Information). These results demonstrate that MLTCs significantly promote tumor metastasis and tumor growth. Immunohistochemistry results showed that, compared with the control group, the intratumor‐injected MLTCs group had higher Ki67, Vimentin, Twist1, and VEGFR1 scores and lower E‐cadherin scores, indicating that MLTCs promoted the proliferation of tumor cells and induced epithelial‐mesenchymal transformation (EMT) of tumor cells (Figure S6, Supporting Information). Meanwhile, the intratumor‐injected MLTCs group had stronger CD11b and Gr‐1 signaling expressions than the control group (Figure S5D‐E, Supporting Information). To test whether intratumor‐injected MLTCs had an inhibitory effect on T cells, flow cytometry analysis showed that the CD8^+^ cell content in the intratumor‐injected MLTCs group decreased from 7.82% to 4.26%, and the CD4^+^ cell content decreased from 15.1% to 11.1% compared with the control group (Figure S5F,G, Supporting Information). Immunofluorescence results showed a significant decrease in the CD3 signal in the intratumorally injected MLTCs group (Figure S5H,I, Supporting Information). These results prove that MLTCs have an inhibitory effect on T cells.

### 2.4 Rbfox3 is a Key Gene for the Transformation of B16 Cells into MLTCs

**Figure 3:**
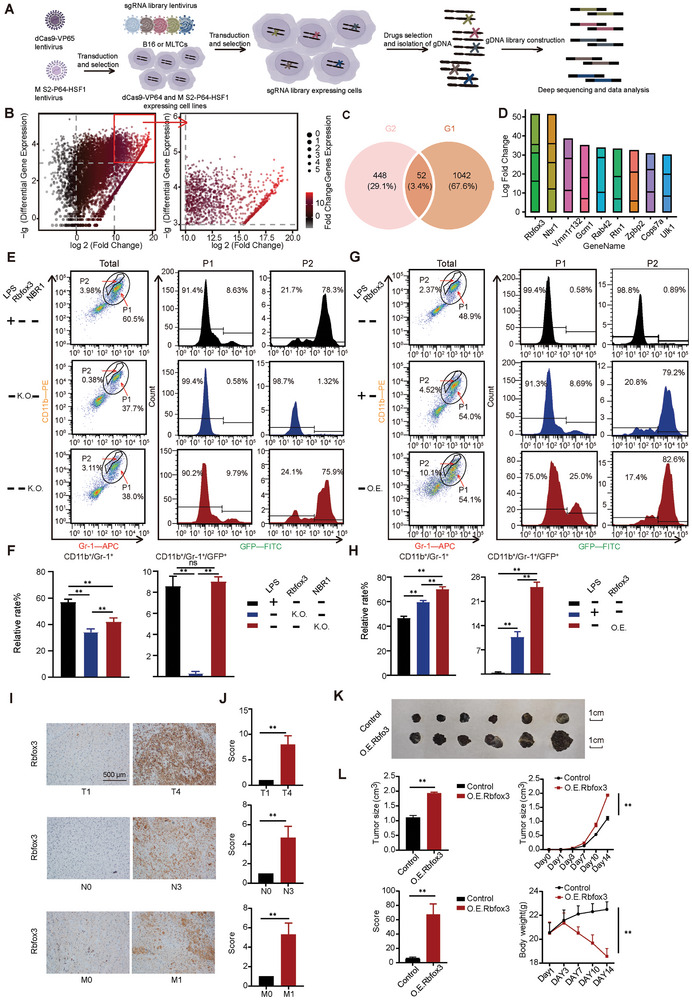
Rbfox3, a key gene inducing the transformation of B16 into MLTC and accelerating tumorigenesis, was found through CRISPR–Cas9 genetic screening. A) Schematic of the CRISPR–Cas9 active screening pipeline. B) Genes screened through positive screening presented in the form of volcano plots. Genes with log2(Fold Change) > 10 and lg(Differential Gene Expression) > 10 considered as the genes highly expressed in MLTCs. C) Filtering of the top 500 ranked genes for Mageck and 1094 highly expressed and significantly differentially expressed genes yielded 52 genes. D) Screening was performed by the number of sgRNAs corresponding to the 52 genes, nine excellent gene‐corresponding sgRNAs with sgRNAs larger than 3 were displayed as stacked columnar plots. E) Gr‐1 and CD11b signals in Rbfox3 knockout or Nbr1 knockout cells were assessed by flow cytometry, and CD11b/Gr‐1 double‐positive cells were again gated to detect GFP signals. F) CD11b, Gr‐1, and GFP positive signal cells were quantified. Statistical analysis was performed, and the results were presented as histograms. G) Flow cytometry was used to detect CD11b and Gr‐1 signals in tumor‐bearing mice in different treatment groups, and CD11b/Gr‐1 double‐positive cells were gated again to detect GFP signals. H) CD11b, Gr‐1, and GFP positive signal cells were quantified and the results were presented as histograms. I) Immunohistochemical analysis of Rbfox3 in melanoma from clinical patients across different pathological stages. J) Quantitative analysis of Rbfox3 immunohistochemical images across different groups. K) Representative tumor images of O.E. *Rbfox3*‐B16‐tumor‐bearing mice. L) Histograms and line graphs depicting tumor volumes on day 14. Additionally, line charts showing body weight and mouse status scores (calculated as body weight × body temperature × hair condition × activity level, with each item scored 1–3 points) over the same period. All values are presented as the mean ± SD, n = 6, ns = not significant, **p < 0.01.

CRISPR screening was used to analyze the molecular mechanism by which LPS promotes the transformation of B16 cells into MLTCs. Normal B16 cells and MLTCs were collected. CRISPR–Cas9 active treatment was performed in the B16 cells and MLTCs (**Figure** **3**A). A total of 1094 genes (G1) were screened based on their fold changes and differential gene expression (Figure 3B). The top 500 genes (G2) were selected using the Mageck RRA algorithm. 52 genes were filtered out by comparing G2 with G1 using Venn analysis (Figure 3C). Nine additional genes were identified based on the number of sgRNAs: *Rbfox3*, *Nbr1*, *Vnm1r132*, *Gcm1*, *Rab42*, *Rtn1*, *Zpbp2*, *Cops7a*, and *Ulk1* (Figure 3D). By examining the differential expression of sgRNAs corresponding to these nine genes, we found that the most differentially expressed sgRNAs and highest number of sgRNAs corresponded to *Rbfox3* and *Nbr1* (Figure 3D). Therefore, we speculated that *Rbfox3* and *Nbr*1 are likely key genes affecting the transformation of B16 cells into MLTCs. We tested this hypothesis by establishing a B16‐EGFP tumor‐bearing model using normal B16‐EGFP cells, *Rbfox3* knockout B16‐EGFP cells, or *Nbr1* knockout B16‐EGFP cells. After LPS induction (Figure 1E), the tumor cells were analyzed by flow cytometry. The P1 population in the tumors of the two knockout groups decreased. The *Nbr1* knockout group P1 cells expressed GFP, whereas the *Rbfox3* knockout group P1 cells lacked GFP expression (Figure 3E,F). These findings illustrate that the loss of the *Rbfox3* gene deprives the ability of B16 cells to transform into MLTCs. *Rbfox3* is the key gene for LPS‐induced transformation of B16 cells into MLTCs. The qPCR and Western Blot results demonstrated that B16 cells highly expressed *Rbfox3* after LPS induction (Figure S7A,B, Supporting Information). *Rbfox3* over‐expression combined with LPS stimulation (*Rbfox3*/LPS) B16‐EGFP tumor‐bearing model was used to evaluate the effect of *Rbfox3* on MLTCs. The model showed significantly increased tumor volumes, reduced body weights, and reduced mouse status scores compared to the control group (Figure S7C‐D, Supporting Information). Subsequently, we sorted the intratumoral CD11b/Gr‐1 double‐positive cells and found that the cellular content of the GFP signal in the intratumoral P1 cells of the *Rbfox3*/LPS treated group was considerably higher than that in the LPS‐treated group. The proportion of GFP‐positive MLTCs (≈25%) in P1 cells of the *Rbfox3*/LPS treated group was more than 3‐fold higher than that (8.69%) in the LPS‐treated group (Figure 3G,H). Consistent with the flow cytometry results, sections from the *Rbfox3*/LPS treated group showed higher CD11b and Gr‐1 positivity than those from the other groups (Figure S7E,F, Supporting Information). Immunohistochemical results for Ki67, Vimentin, Twist1, and VEGFR1 indicated higher malignancy in the *Rbfox3*/LPS treated groups than in the control group (Figure S8, Supporting Information). We further analyzed the expression of Rbfox3 protein in tissue samples across different pathological stages using immunohistochemistry (Figure 3I,J). The results showed that high expression of Rbfox3 was positively correlated with the degree of malignancy at the clinicopathological stage, which was consistent with our conclusion. We also established an O.E. *Rbfox3*‐B16 cells tumor‐bearing mice model to evaluate the effect of Rbfox3 on tumors. Over‐expression of Rbfox3 resulted in a substantial increase in tumor volume, almost doubling compared to that in the control group, and a significant decrease in the survival score of the mice (Figure 3K,L). The above experiments showed that *Rbfox3* is a key gene for the transformation of B16 cells into MLTCs, which affects the malignant prognosis of tumors in mouse models, and that the expression level of Rbfox3 is closely related to clinical patient prognosis.

### 2.5 *Rbfox3* Transcription Regulates Ass1 Expression and Drives MLTCs Formation

**Figure 4:**
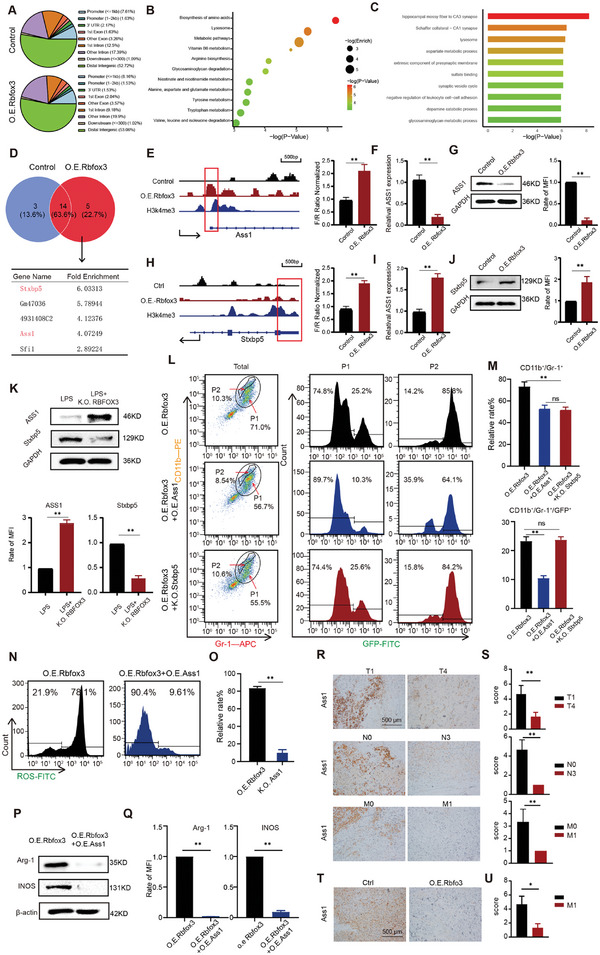
Rbfox3 transcription regulates Ass1 expression and drives MLTCs formation. A) Resulting peaks at various locations along the genome in the control and Rbfox3 overexpressing groups after the cut and run experiment and percentage occupied. B‐C) Venn diagram of 166 genes associated with the post cut and run of overexpressed Rbfox3 versus 155 genes associated with the post cut and run of the control, GO analysis, and KEGG analysis of 77 genes unique to the overexpressed Rbfox3 group. D) Venn diagram of the two groups of bound promoters showing genes specifically bound by overexpressed Rbfox3 and demonstrating their enriched expression. E) Chip SEQ showing that Ass1 binding was enhanced in the overexpressed Rbfox3 group compared with in the control group. F‐G) qPCR and Western Blot data showing reduced Ass1 mRNA and protein levels in the Rbfox3 overexpressing group. H) Chip SEQ revealed enhanced binding to Stxbp5 in the group overexpressing Rbfox3 relative to controls. I‐J) qPCR and Western Blot data showing increased Ass1 mRNA and protein levels in the Rbfox3 overexpressing group. K) Protein levels of Ass1 and Stxbp5 after Rbfox3 knockdown were detected using Western Blot analysis and presented as histograms. L–M) Flow cytometry was used to detect the CD11b and Gr‐1 signals of tumor‐bearing mice in different treatment groups, and CD11b/Gr‐1‐double‐positive cells were gated again to detect GFP signals. N‐O) ROS levels were detected by flow cytometry, statistical analysis was performed and the results were presented as histograms. P‐Q) Western Blot analysis was performed to detect the changes in the expression levels of iNOS and Arg‐1. The results of statistical analysis were presented as histograms. R‐S) Immunohistochemical analysis of Ass1 in melanoma across different pathological stages. Quantitative analysis of Ass1 expression. T‐U) Immunohistochemical analysis of Ass1 in O.E. Rbfox3‐B16 tumors and the results were presented as histograms. All values are presented as the mean ± SD, n = 6, ns = not significant, **p < 0.01.

Normal B16 cells and over‐expressing *Rbfox3* groups were subjected to cut and run experiments to further explore the molecular mechanism by which MLTCs exert immunosuppressive effects and promote tumor development. Two sets of sample data were collected (**Figure** **4**A) and subjected to differential analyses. Specific genes in the O.E. *Rbfox3* group were subjected to KEGG and GO analyses (Figure 4B,C). Several genes were differentially upregulated by the promoter in the O.E. *Rbfox3* group (Figure 4D). Normal B16 cells and MLTCs induced by Rbfox3 over‐expression were subjected to a chromatin immunoprecipitation (ChIP) test, and the differences between the Rbfox3 protein‐DNA binding complexes of the two groups were compared by sequencing. The Chip‐seq data showed strong peak signals in the promoter regions of *Ass1* and *Stxbp5* in the O.E. *Rbfox3* group that was verified by a dual‐luciferase assay (Figure 4E,H). Subsequently, we detected the mRNA and protein contents using qPCR and Western Blot analysis and found that the content of Stxbp5 in the O.E. *Rbfox3* group increased (Figure 4I,J). However, the mRNA and protein levels of Ass1 decreased (Figure 4F,G). These findings illustrated that Rbfox3 acts as a transcription factor that enhances binding to *Ass1* and *Stxbp5* promoters and activates the transcription and translation of Stxbp5. However, the expression of mRNA and protein of Ass1 are inhibited by Rbfox3 over‐expression. By comparing the amounts of Ass1 and Stxbp5 in the K.O. *Rbfox3* group under LPS stimulation, we found that the content of Ass1 protein increased and Stxbp5 protein decreased in the K.O. *Rbfox3* group (Figure 4K). This finding is consistent with the experimental results. We designed O.E. *Rbfox3* group, O.E. *Rbfox3* + O.E. *Ass1* group, and O.E. *Rbfox3* + K.O. *Stxbp5* groups to clarify whether *Ass1* or *Stxbp5* are the key genes for MLTCs formation. Flow cytometry analysis showed that the GFP signal of P1 cells in the O.E. *Rbfox3* + O.E. *Ass1* group decreased, whereas the GFP signal of P1 cells did not decrease in the O.E. *Rbfox3* + K.O. *Stxbp5* group. These findings illustrate that Ass1 is a key gene that affects the generation of MLTCs, whereas Stxbp5 does not (Figure 4L,M). Ass1 is a key enzyme in arginine biosynthesis, and in patients with cancer, low Ass1 expression in tumor tissues is associated with poor prognosis.^[^ ^28^ ^]^ We took O.E. *Rbfox3* as the control group and O.E. *Rbfox3* + O.E. *Ass1* as the experimental group. We discovered that following Ass1 overexpression, the ROS content decreased significantly from 78.1% to 9.61% (Figure 4N,O). Western Blot analysis confirmed that the levels of iNOS and Arg‐1 decreased significantly after Ass1 overexpression (Figure 4P,Q). These experiments confirmed that a reduction in Ass1 increased ROS, iNOS, and Arg‐1 contents after Rbfox3 over‐expression. We analyzed the expression of the Ass1 in tissue samples across different pathological stages, and the results showed that the expression of Ass1 was negatively correlated with the degree of malignancy at the clinicopathological stage (Figure 4R,S). We established an O.E. *Rbfox3*‐B16 cells tumor‐bearing mice model, and immunohistochemical staining of O.E. *Rbfox3* tumor tissues revealed suppressed Ass1 expression in tumors with high Rbfox3 expression (Figure 4T,U). Both clinical and animal experiments have shown that Ass1 plays an important role in the malignant evolution of tumors.

### 2.6 Rbfox3 Inhibits the Expression of Ass1 Protein through Phase Separation

**Figure 5:**
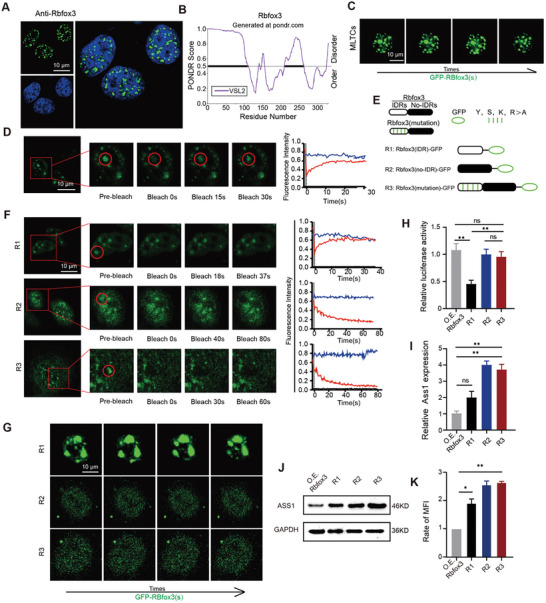
Rbfox3 inhibits the expression of Ass1 protein through phase separation. A) Immunofluorescence experiments were performed to detect MLTCs, and representative images were captured by confocal microscopy. B) Diagram of Rbfox3 IDR predicted by the website (PONDR vsl2). C) Representative time‐series images of the live cell fluorescence microscopy of lipid droplet fusion. D) Partial FRAP experiments were performed, and line graphs were used to present fluorescence intensity in images. E) Schematic diagram of the plasmid design for R1, R2, and R3. F) Partial FRAP experiments on groups R1, R2, and R3 were performed, and line graphs were used to present fluorescence intensity in images. H) Representative time‐series cyto‐fluorescence microscopy images of lipid droplet fusion. G) Luciferase reporter assay showing the binding ability of different groups to the Ass1 promoter. H) qPCR shows the amount of *Ass1* mRNA expression in different groups. I‐K) Ass1 protein levels were measured using Western Blot. Statistical analysis was performed, and the results were presented as histograms. All values are presented as the mean ± SD, n = 6, ns = not significant, *p < 0.05, **p < 0.01.

Immunofluorescent images of Rbfox3 in MLTCs showed that Rbfox3 can form discrete spots in the nucleus (**Figure** **5**A). A growing body of evidence indicates that phase‐separated proteins readily form droplets in vivo,^[^ ^29^ ^]^ and proteins that form bright foci often have an intrinsic ability for phase separation.^[^ ^30^ ^]^ The proportion of IDR in proteins can be used to predict whether the overexpression of Rbfox3 can form phase separation nuclear condensates. The PONDR Score above 0.5 is considered a marker of IDR. The regions of Rbfox3 with a PONDR Score above 0.5 account for 45.29% (Figure 5B). Rbfox3 in MLTCs was continuously visualized using a live cell fluorescence microscope, thus successfully capturing the droplet fusion process (Figure 5C). One large Rbfox3 droplet was subjected to the FRAP analysis. We found that the brightness of the Rbfox3 fluorescence rapidly recovered to more than 85% of the original fluorescence in the partially quenched region within 30 s (Figure 5D), indicating that the Rbfox3 protein from MLTCs is highly mobile and has phase separation properties. We divided the Rbfox3 protein into an intrinsically disordered region (IDR) and a non‐intrinsically disordered region (no‐IDR) to further confirm the role of each part of the Rbfox3 protein (Figure S9A, Supporting Information). We further designed four protein mutants, namely, Rbfox3 (IDR)‐GFP, Rbfox3 (no‐IDR)‐GFP, and Rbfox3 (mutation)‐GFP, and used the GFP monomer as the tracer protein (Figure 5E).

R1, an Rbfox3 (IDR)‐GFP protein, was designed to verify the function of the IDR region. It consists of the IDR sequence of Rbfox3 and the sequence of the GFP gene. R2, the Rbfox3 (no‐IDR)‐GFP protein, was designed to study the function of the no‐IDR region. It consists of the no‐IDR gene sequence of Rbfox3 and the sequence of the GFP gene sequence. The amino acid composition, especially the serine (s), tyrosine (y), arginine (R), and lysine (k) residues, of the Rbfox3 IDR region was highly conserved between humans and mice (Figure S9C, Supporting Information). These amino acids are critical for the phase separation of several proteins.^[^ ^31^, ^32^ ^]^ We designed R3, the Rbfox3 (mutation)–GFP sequence, which is composed of GFP linked by the mutated IDR region (all conserved S, Y, R, or K residues were replaced by alanine), to identify the key amino acids in Rbfox3‐IDR for phase separation.

Subsequently, the above three groups of samples were continuously observed under live‐cell fluorescence microscopy. We found that only the R1 group presented obvious droplet phenomena and that droplet fusion could occur over time (Figure 5F). Moreover, the FRAP experiments demonstrated that the P1 group recovered after fluorescence quenching, whereas the R2 and R3 groups did not (Figure 5G). This result confirms that the IDR in over‐expressed Rbfox3 is indeed the key domain that causes phase separation. Subsequently, a luciferase reporter assay was performed to determine the binding abilities of the different groups to the Ass1 promoter. Only R1 showed lower expression level (Figure 5H), indicating that the no‐IDR of Rbfox3 is the key domain for binding with the Ass1 promoter. qPCR and Western Blot analyses showed that the R2 and R3 groups had the highest mRNA and protein expression levels, while the R1 group also had higher mRNA and protein expression levels than the O.E. *Rbfox3* group (Figure 5I–K). These results indicate that the no‐IDR is the key domain for binding to the *Ass1* promoter, promoting the transcription and translation of Ass1. The IDR is the critical region for phase separation, which does not affect the binding of Rbfox3 to the *Ass1* promoter but inhibits the mRNA and protein expression of Ass1.

### 2.7 Crocin Targets Rbfox3, Interfere the Formation of Rbfox3 Phase Separation Particles, and Effectively Inhibits the Generation of MLTCs

**Figure 6:**
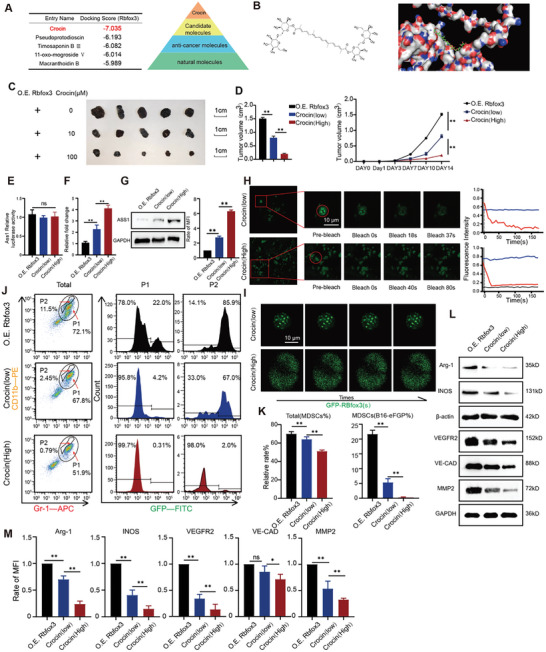
Crocin binds to Rbfox3 to suppress MLTCs generation, regulating the tumor immune microenvironment. A) Screening of natural drug small molecules that can bind to Rbfox3 through docking strategies. B) Rbfox3 protein structure for crocin binding and crocin chemical structure. C) Representative tumor images of O.E. *Rbfox3*‐B16 tumor‐bearing mice treated with different concentrations of crocin. D) Histograms and line graphs depicting tumor volumes on day 14. E) Dual luciferase reporter gene assay was performed to examine the effect of different concentrations of crocin on the promoter binding abilities of Ass1. F) qPCR was performed to detect the effect of different concentrations of crocin on *Ass1* mRNA expression levels. G) Western Blot analysis was used to detect the levels of protein expression, which were presented as histograms after quantification. H) Partial FRAP experiments were performed on groups treated with low or high concentrations of crocin, and line graphs present the fluorescence intensity in images. I) Representative time‐series images of live cell fluorescence microscopy of lipid droplet fusion in groups under treatment with low and high concentrations of crocin. J‐K) Flow cytometry was performed to detect CD11b and Gr‐1 signals in tumor‐bearing mice, and CD11b/Gr‐1‐double‐positive cells were gated again to detect GFP signals. L‐M) Protein levels of iNOS and Arg‐1 were detected using Western Blot analysis. All values are presented as the mean ± SD, n = 5, ns = not significant, *p < 0.05, **p < 0.01.

Previous studies showed that Rbfox3 plays a crucial role in modulating MLTCs generation. The repression of the *Rbfox3* may be a useful target inhibiting the generation of MLTCs. So we wanted to identify a molecule that inhibits the transcriptional inhibition activity of *Rbfox3* on Ass1 and immunosuppressive effects of MLTCs. Natural drug molecules were screened for the structure of the Rbfox3 protein by molecular docking simulation, and their complementarity values were scored by the binding site (**Figure** **6**A). After screening for monomers with scores greater than 5.0, we found that crocin had the strongest inhibitory effect on MLTCs in vivo (Figure 6B). Subsequently, low (10 µM) and high (100 µM) concentrations of Crocin were injected into tumor‐bearing mice. The tumor volume decreased significantly (Figure 6C,D). Three groups of tumor cells were used in the fluorescence double reporter assay, and Rbfox3 binding to the Ass1 promoter was unaffected in the treated group (Figure 6E). However, qPCR and Western Blot analyses demonstrated that crocin treatment significantly increased the mRNA level and protein content of Ass1 (Figure 6F,G). Crocin reverses the transcriptional repression of Ass1 by Rbfox3. We found that the phase separation property of over‐expressed Rbfox3 cells also disappeared in the group treated with a high concentration of crocin. Time‐series live cell fluorescence microscopy revealed that the experimental group could not fuse to form bright droplets, and in the FRAP experiment, the fluorescence intensity could not be recovered (Figure 6H,I). Taken together with previous conclusions, these results suggest that crocin does not affect the promoter binding of Rbfox3 to Ass1, but affects the formation of phase separation particles. Crocin mainly affect Rbfox3 phase separation. We further subjected tumor cells to flow cytometry to detect MLTCs and verify the relevant functions of crocin. We found that with the increase in crocin concentration, the content of MDSCs decreased significantly, and the MLTCs almost disappeared under treatment with a high concentration (100 µM) of crocin (Figure 6J,K). This finding showed that crocin not only reduced the number of MDSCs in tumors, but also inhibited the production of MLTCs. These results were confirmed by Western Blot. In contrast to the control treatment, crocin treatment resulted dose‐dependent reduction in Arg‐1, iNOS, VEFGR2, VE‐Cadherin, and MMP2 (Figure 6L,M).

### 2.8 Crocin Combined with PD‐1 Antibody Therapy can Reduce MLTCs and Effectively Inhibit the Malignant Progression of Melanoma

We designed the experiment to detect the content of immune checkpoint labeled PD‐L1 on MLTCs. Compared with the control group, the expression level of MLTCs was significantly increased (Figure S10A,B, Supporting Information). Given that the programmed death protein PD‐1 plays an important role in suppressing immune responses and promoting self‐tolerance by regulating the activity of T cells, activating the apoptosis of antigen‐specific T cells, and inhibiting the apoptosis of regulatory T cells.^[^ ^33^ ^]^ Through flow analysis, we found that the content of P1 cells decreased in the αPD‐1 (PD‐1 antibody) treated group alone, while the GFP signal of P1 cells did not change, indicating that the production of MLTCs was not inhibited. Although crocin alone inhibited the generation of MLTCs, the MDSCs content remained high, and the combination group exhibited an extremely strong ability to inhibit P1 and MLTCs (Figure S10C,D, Supporting Information). Compared with the control group, the number of MDSCs in the experimental group decreased by nearly 40%, from 66.1% to 28.2%. Moreover, the MLTCs were completely inhibited. The tumor volume in tumor‐bearing mice in the combination group was significantly reduced (Figure S10E,F, Supporting Information). Immunohistochemical analyses were performed. Compared with the control group, the Ki67, Twsit1, and VE‐cadherin scores of the groups treated with crocin or αPD‐1 alone decreased, and the scores of the combined drug group were significantly lower (Figure S10G,H, Supporting Information). The combination of crocin and PD‐1 can effectively inhibit the formation of MLTCs and tumor growth, and has a better antitumor effect than each drug alone.

## 3 Discussion

In this study, we found that tumor cells could be transformed into MLTCs to mimic MDSCs under LPS stimulation, thereby reshaping the immunosuppressive TME. Our study also elucidates the mechanisms underlying the transformation of melanoma cells into MLTCs. We demonstrated that some tumor cells could transform into MDSC‐like cells. In LPS simulated chronic inflammatory microenvironment This work not only revealed new possibilities for the origin of immunosuppressive cells but also verified the high plasticity of tumor cells.

Unlike in the normal environment, PMN‐MDSCs in the TME are strongly immunosuppressive. Similar to PMN‐MDSCs, the transformation of MLTCs depends on TME with chronic inflammation. MLTCs and PMN‐MDSCs express the key markers CD11b and Gr‐1. In terms of function, the presence of MLTCs significantly promoted tumor growth in tumor‐bearing mice, and promoted the occurrence and development of tumors. In vitro, MLTCs inhibit the immune activity of NK cells and induce the apoptosis of immune cells, such as T cells. Moreover, they promote tumor cell proliferation, migration, vascular mimicry, and other malignant evolutionary processes. These functions are very similar to those of PMN‐MDSCs in the TME. We hypothesized that some PMN‐MDSCs may be originally derived from tumor cells. Similar to immunosuppressive cells, the biological functions of MLTCs include immunosuppression and tumor growth promotion. However, Compared with tumor cells, MLTCs showed weaker proliferative function and adhesion. This semi‐adherent property may explain why MLTCs exist only in tumors and do not enter the bone marrow and spleen with the peripheral blood.

MDSCs can inhibit immune cell and T cell activity. L‐arginine can produce citrulline and nitric oxide (NO) based on iNOS or NOS2 or is converted into urea and L‐ornithine by arginase. This effect may lead to the loss of CD3 expression and damage T cell function. NO produced by iNOS‐expressing MDSCs was sufficient to block T cell responses. Our study revealed that the immunosuppressive effects of MLTCs were closely related to argininee expression. Under LPS stimulation, Rbfox3 can inhibit the expression of Ass1 and arginine synthesis. Ass1 catalyzes the formation of argininosuccinate from citrulline and aspartate.^[^ ^28^ ^]^ In cancer patients, low Ass1 expression in tumor tissues is associated with poor prognosis.^[^ ^34^ ^]^ The decreased expression of Ass1 not only plays an immunosuppressive role but also affects the production of MLTCs.

We discovered that the Rbfox3 protein underwent phase separation in the nucleus. In biology, phase separation is a phenomenon in which biological molecules form liquid‐like aggregates (also known as membrane‐free compartments or coacervates) that have different physical and biological functions from freely soluble macro‐molecules.^[^ ^35^, ^36^ ^]^ These membrane‐free compartments can be assembled by using highly mobile proteins, nucleic acids, and other molecules.^[^ ^37^ ^]^ Given the absence of membranes, the components in membrane‐free compartments can move quickly and mutually transform to reach the chemical equilibrium of various subcomponents and maintain the formation of droplets.^[^ ^38^ ^]^ Protein IDRs play important roles in aggregation. IDR is a protein sequence that lacks 3D structural stability, and its structural heterogeneity allows it to interact in multiple directions to form droplets. Disordered sequences are also a prerequisite for determining whether a protein can undergo phase separation, and highly conserved amino acid residues in the IDR of a protein are key to phase separation.^[^ ^39^ ^]^ Phase separation, resulting in aggregate formation, plays an important role in numerous cellular processes, including transcriptional regulation of DNA, control of signal transduction, ubiquitination of proteins, division of cells,^[^ ^40^, ^41^, ^42^, ^43^ ^]^ and transport of nuclear pore complexes.^[^ ^44^ ^]^ Biological macromolecules formed by phase separation also participate in the development of various diseases,^[^ ^45^ ^]^ including neurodegenerative diseases^[^ ^46^ ^]^ and cancer.^[^ ^47^ ^]^

Rbfox3, also known as NeuN, is generally believed to exist in the nuclei of mature neurons, and is considered a clear marker of mature neurons.^[^ ^48^, ^49^ ^]^ However, in some cancers, the tumor cells of patients with high Rbfox3 expression show high tumorigenicity and stem cell‐like characteristics with self‐renewal and pluripotent differentiation potential. The Rbfox3 protein is divided into IDR and no‐IDR proteins in accordance with its amino acid sequence. The Rbfox3 sequence is highly conserved among species. Further research revealed that an IDR composed of highly conserved amino acid sequences endows Rbfox3 with phase separation characteristics. No‐IDRs can combine with the promoters of Ass1 and Stxbp5 to participate in transcriptional regulation. IDR induces proteins to form phase separation particles and inhibits the expression of Ass1 mRNA and proteins. After crocin affects Rbfox3 phase separation, the transcriptional inhibition of Ass1 is reversed. This study provides new insights for future drug research and development.

A growing body of evidence has shown that some tumor cells are highly plastic. In the TME, cancer cells are affected by a complex environment and have abilities similar to those of embryonic stem cells. We also found that tumor cells could transform into MLTCs in the TME during chronic inflammation. This study not only supplements the available evidence on the variability of tumor cells, but also presents the possibility of different sources of immunosuppression cells for the first time. The no‐IDR region of Rbfox3 can bind to the Ass1 promoter and the IDR region of Rbfox3 inhibit the mRNA and protein levels of Ass1 through phase separation, thus driving the production of MLTCs. Crocin is a water‐soluble carotene with a potent pharmacological activity that inhibits the production of ROS and suppresses the production of proinflammatory cytokines.^[^ ^50^ ^]^ In addition, crocin has anti‐migratory, anti‐invasive, and anti‐vasculogenic mimicry capabilities, can substantially attenuate tumor ECM adhesion, and is a potential agent against cancers,^[^ ^51^ ^]^ such as breast cancer.^[^ ^52^ ^]^ Crocin can inhibit the production of MLTCs by targeting Rbfox3 and improve the anti‐tumor effect of the PD‐1 antibody, which is expected to be developed as an anti‐tumor drug.

## 4 Experimental Section

### Cell Culture and Stable Cell Lines

B16 and B16‐EGFP cells were purchased from KeyGen Biotech (Nanjing, China). B16 and B16‐EGFP cell lines were cultured in a 1:1 ratio of 1640 medium (Keygen Biotech, 1640 with 20% FBS and 2% penicillin–streptomycin) to DMEM (Gibco, Grand Island, NY, USA, DMEM with 20% FBS and 2% penicillin–streptomycin). MDSCs were derived in vitro from the femur and tibia bone marrow cells of C57/BL/6J mice. The bone marrow was isolated from donor mice euthanized by cervical spine dislocation. The bone marrow was flushed with sterile PBS, and red blood cells were lysed with a hypotonic saline solution after the bone marrow was isolated. After centrifugation at 1000 rpm, the supernatant was removed, the precipitate was resuspended, and a flow sorter (BD Aria III) was used to sort the MDSCs.

### In Vitro B16 Cells Transformation

First, freshly obtained tumor tissue was placed in a petri dish containing cold PBS and gently shaken to wash away blood and impurities from the surface. After cutting the tissue into small pieces, it was homogenized into a uniform suspension using an electric homogenizer. The homogenized mixture was then centrifuged at 4 °C and 3000 RPM for 30 minutes. The supernatant was collected to obtain the tumor extract. The BCA protein quantitative kit A55860 (500 mL) was used to generate a standard curve. Solutions of bovine serum albumin (BSA) were prepared at concentrations of 0, 0.2, 0.4, 0.6, 0.8, and 1.0 mg mL^−1^ to react with Bradford's reagent. The absorbance of these solutions was measured at a specific wavelength (usually 595 nm) to establish the standard curve. Finally, an appropriate amount of tumor extract was reacted with the Bradford reagent and measured the absorbance at the same wavelength. Using the standard curve, the protein content in the tumor extract was calculated. A total of 5.2 × 10^5^ B16 cells were seeded in 6 cm culture dishes, induced by adding 1µg mL^−1^ LPS (Sigma‐Aldrich, Co. St. Louis, MO, USA, 1 mg mL^−1^) and 1 mg mL^−1^ tumor extract, which was added every day, other culture conditions were the same as the conditions mentioned above and differentiated into CD11b^+^ and Gr‐1^+^ cells after 15 days.

### In Vivo MLTCs Induction of Tumor by LPS

Tumor growth studies were initiated in C57BL/6 mice by intradermal injection of 2×10^6^ B16‐EGFP cells suspended in 200 µL of PBS. For LPS injection, animals were injected (intratumoral injection) with 4 mg kg^−1^ LPS in PBS. Injections were administered every alternate day until the endpoint was reached. First, the tumor tissue was fully cut up, and 5–6 times the amount of tissue was added to the enzymolysis solution, which was digested in a 37 °C shaking table for 4–6 h (10 mL enzymolysis solution formula: 0.2% collagenase, Sigma SCR103, 0.01% hyaluronidase, Solarbio, H8030, DMEM, GIBCO, NY, USA), Prepare the tumor tissue into a single‐cell suspension, then seed the cells into BeyoGold™ ultra‐low attachment 100 mm culture dishes and let them stand for 4 h. At this time, collecting all the cells in the supernatant can screen out most of the tumor cells attached to the wall. In the second step, after cleaning with PBS, cell counting was performed with a cell counting board (Watson 177‐112C) with no more than 10^8^ cells per 100 µL. 0.625 µL CD11b (invitrogen, 12‐0112‐81), 0.625 µL Gr‐1 (invitrogen, 17‐5931‐81) flow antibody. Dye at 37 °C for 40 minutes. Then, obvious dead cells and cell discretion were excluded due to voltage anomaly by using forward Angle (FSC) and lateral Angle (SSC). MDSCs cells were screened by Gr‐1 and CD11b, the gating strategy followed by identifying CD11b and Gr‐1 positive areas based on three control groups: isotype control antibody, only CD11b (without Gr‐1), and only Gr‐1 (without CD11b). Finally, the GFP positive area was delineated using GFP negative and GFP positive control groups. GFP signals were screened from Gr‐1/CD11b double‐positive cells. FACS experiments were performed on a BD Aria III cell sorting system to obtain the MLTCs.

### Lung Metastasis and Lung Tumor Models

For lung metastasis models, cells (2 × 10^6^) in 200 µL PBS were injected into the tail vein of C57BL/6 mice. After euthanasia, the metastatic nodules on the surface of the lung lobes were manually counted. Lung nodules measuring >1 mm in diameter were counted. All animal experiments were approved by the Institutional Ethics Committee. All experiments were performed on 7–12‐week‐old male C57BL/6 mice, unless otherwise specified. The mouse was housed in a specific pathogen‐and viral antibody‐free animal facility.

### Immunohistochemical (IHC) Staining

Paraffin‐embedded sections were dewaxed with xylene, treated with graded ethanol, and rehydrated in phosphate‐buffered saline. The slides were incubated in sodium citrate antigen retrieval solution at 98 °C for 20 min and blocked with 5% goat serum for 2 h. After blocking, sections were incubated in 0.3% hydrogen peroxide for 24 h. Sections were incubated overnight with the following primary antibodies: Ki67, E‐Cadherin, Vimentin, Twist‐1, VE‐Cadherin, and VEGFR2. Sections were incubated with an immunopotentiator (Affinity Bioreagents, Golden, Co, USA) for half an hour, followed by half‐hour incubation with an anti‐mouse/anti‐rabbit immunoperoxidase polymer. The sections were stained with hematoxylin for 9 min. After staining, the sections were dehydrated, mounted, and observed by immunohistochemical staining using a microscope (Nikon, Tokyo, Japan). IHC results were scored using a semi‐quantitative scoring scale, and the staining intensity and positive area were recorded. The staining index (values: 0–3) was calculated by multiplying the intensity of tissue primary antibody‐positive staining (negative, 0; weak, 1; moderate, 2; strong, 3) by the proportion of immunopositive cells of interest (<30%, 1; 30–60%, 2; 60–100%, 3). The sections were stained using an HE staining kit (Solarbio), dewaxed with xylene, treated with graded ethanol, and rinsed with tap water. The sections were stained with hematoxylin for 9 min and then stained with eosin for 2 min. After staining, the sections were dehydrated, mounted, and observed by HE staining using a microscope (Nikon, Tokyo, Japan).

### Scratch Test and Transwell Invasion Assay

The B16 cells were assayed for their ability to migrate and proliferate using a scratch assay. The B16 cells were seeded in 24‐well plates and grown to confluence. Cells were scraped with a 200 µL pipette tip, wounds were formed, and cultured in the serum‐free medium using a live cell microscope. (Nikon) Take pictures every 12 h, measure the scratch distance, and use image J to judge the results. The invasive ability of the cells was determined using a transwell assay. For invasion assays, add 5 × 10^4^ cells in 200 µL of serum‐free medium to the upper chamber coated with Matrigel (San Jose, CA, USA). Then, add 800 µL of medium containing 10% FBS to the bottom chamber. After 24 h of culture, the cells on the lower surface of the filter were fixed with 4% paraformaldehyde (PFA; Solarbio), and the invasive cells were counted using crystal violet staining.

### FACS Analysis

For each flow tube, prepare 1 × 10^6^ cells, incubate them with the corresponding direct‐labeled antibody in the dark for 2 h according to the experimental purpose, centrifuge at 800 rpm after staining, washed with PBS, passed through a 400‐well mesh, filtered and added to flow tubes, obvious dead cells and cell discretion were first excluded due to voltage anomaly by using forward Angle (FSC) and lateral Angle (SSC). In the second step, MDSCs cells were screened by Gr‐1 and CD11b. In the third step, GFP signals were screened from Gr‐1/CD11b double‐positive cells, analyzed with a flow cytometer (easyCyte HT, Millipore, USA), and data analyzed with FLOW JO software. In the context of flow cytometry analysis, a detailed gating approach is employed to obtain MDSCs based on their physical properties (FSC/SSC) and specific marker expression (CD11b/Gr‐1), and P1 and P2 are distinguished by differences in fluorescence intensity. Once the cell populations are clearly delineated, the number of cells within each population (P1 and P2) is quantified. Subsequently, the ratio of P1 cells to P2 cells (P2/P1) is calculated for each experimental group. Statistical differences in the P2/P1 ratios among different groups are then assessed.

### Cells and Tissue Immunofluorescence Preparation and Staining

The B16 cells were seeded in 24‐well culture plates and plated on cell‐climbing slices. cells (MDSCs) were plated on glass slides. The cells were mounted with a 4% PFA‐fixed solution. The tissue sections were dewaxed and rehydrated. Sodium citrate was used for antigen repair, and 3% hydrogen peroxide was used to inactivate endogenous peroxidase for 30 min. The sections were subjected to antigen retrieval, blocked with 5% goat serum, and incubated with primary antibodies overnight. Thereafter, the cells were incubated with direct antibodies (CD11b and Gr‐1) diluted in an antibody diluent for 2 h. Tissues and cells were incubated with primary antibodies (Rbfox3, CD3, CD11b, and Gr‐1) overnight at 4 °C, followed by incubation with secondary antibodies for 30 min. Finally, the tissues/cells were stained DAPI (Beyotime), the tissues/cells were stained DAPI.

### Western Blot

First, whole‐cell lysates under different treatment conditions were analyzed by sodium dodecyl sulfate‐polyacrylamide gel electrophoresis (SDS‐PAGE), and then the proteins were transferred to PVD membranes and blocked with 5% skim milk for 2 h. It was blocked with the primary antibody iNOS, TLR4, NF‐κB, Arg‐1, TNF‐α, MyD88, β‐actin overnight at 4 °C, and the corresponding horseradish peroxidase secondary antibody (Affinity, 1:5000) was used for binding and labeling, and finally observed with a gel imaging system (ChemiScope 6000, CLIX, Shanghai, China).

### In Vitro Blood Vessel Formation Assay

A 24‐well plate was coated with Matrigel, and cells (2.5 ×10^5^ cells per well) were seeded on the plate, observed with a live cell imager, and analyzed for the number of tubes with image J.

### Crispr‐Cas9 Active Screening and Results Analysis

Cells were screened using the CRISPR‐Cas9 system, and the CRISPR–Cas9 lentiviral system was purchased from Obio Technology (Shanghai, China), crispr‐cas9. The lentiviral vectors used were pSLenti‐CMV‐CuO‐MS2‐P65‐HSF1‐2A‐Hygro‐WPRE and lentils‐VP64‐BLAST. The lentiviral transfection Vector is the Mouse Cas9 Active Triple Vector library, which contains 23 439 genes. Cells (2 × 10^7^) were infected with the lentivirus library at an MOI (multiplicity of infection) of 0.3 with 8 µg mL^−1^ of polybrene. After 4 days, the cells were infected with the lentivirus library at a MOI of 0.7. Following this, the cells were selected based on their resistance to puromycin. The products were then sent for sequencing at Sequiserve, and the sequencing results were visualized using Snapgene software. All analyses were performed using R (v.4.1.2). R packages such as pheatmap, ggplot2, ggrepel, and gplots were used for figure generation.

### ROS Assay

Cells were plated into six‐well plates, the production of reactive oxygen species in the cells was determined using a reactive oxygen species assay kit (KeyGen Biotech), and fluorescence was measured using a flow analyzer (easyCyte HT, Millipore) FITC channel according to the manufacturer's instructions.

### Clone Formation

To perform colony formation experiments in 6‐well plates, prepare 103 cells, suspend them in normal medium, and incubate them in a constant temperature incubator at 37 °C, 5% CO2 for 14 days. The cells were then fixed in 4% PFA for 20 min. After dissection, stained with crystal violet. A microscope was used to observe the number of colonies, and image J was used for count analysis.

### Carboxy Fluorescein Succinimidyl Ester (CFSE) Staining Assay

CFSE staining kits were purchased from KEYGEN Biotechnology. The cells were stained with Cell Trace CFSE carboxyfluorescein succinimidyl ester (CFSE; Thermo Fisher Scientific) according to the manufacturer's instructions. The cells were then rinsed three times with PBS and analyzed using a flow cytometer (easyCyte HT).

### Duolink In Situ Proximity Ligation Assay

Duolink assays were performed using the Duolink In Situ Red Starter Kit Mouse/Rabbit (Sigma‐Aldrich). Briefly, cells were washed with PBS, fixed with 4% formaldehyde, punched with 0.5% Triton, and blocked with 3% bovine serum albumin at 37 °C for 1 h, cells were incubated with primary antibody overnight at 4 °C, combined with a secondary antibody and PLA probes were combined and incubated at 37 °C in the dark for 60 min and 37 min. The cells were washed three times with the wash buffer. Finally, the cells were stained with DAPI and observed under a microscope.

### CUT and RUN

According to the manufacturer's instructions, CUT and RUN Detection Kits (CST) were used to detect the binding of Rbfox3 to DNA. A total of 10^5^ cells were analyzed under each condition. After washing, the cells were added to concanavalin A beads and incubated for 10 min with rotation to immobilize the cells, which were then collected using a magnetic rack. The supernatant was discarded, and the beads were resuspended in antibody buffer (wash buffer supplemented with 0.08% digitonin (Millipore) and 2 mM EDTA). The antibody (1 µg) was added to the beads, which were incubated overnight at 4 °C with rotation. The following day, the beads were washed twice with digitonin buffer (wash buffer supplemented with 0.08% digitonin). CUTANA pA/G MNase (2.5 µL; EpiCypher, 15–1016) was added to 50 µL digitonin buffer and incubated for 10 min. The beads were washed twice with digitonin buffer, and the MNase was activated with 2 mM CaCl2 and incubated for 0.5 h at close to 0 °C. MNase activity was terminated by adding 100 µL stop buffer. The supernatant was collected and centrifuged at 16 000 × *g* for 5 min. DNA was purified using a DNA purification buffer and spin columns. The products were then sequenced. The results were visualized.

### Luciferase Reporter Assay

Luciferase reporter assay was performed using the Dual‐Luciferase Reporter Assay Kit (Beyotime) according to the manufacturer's protocol. Each experiment was performed in triplicates.

### FRAP Assay

The FARP measurements were performed on the MLTCs. After the cells were transfected with the fusion plasmid (GFP‐Rbfox3) for 24 h, the region of interest was bleached using a confocal laser scanning microscope (Zeiss LSM 800). Fluorescence intensity was recorded before and after bleaching.

### Cell Toxicity Assay by LDH Release

Target cells and effector cells were added to a U‐shaped 96‐well culture plate with 100µL each (effector‐to‐target ratio of 50:1). For the target cells natural release well, 100µL of target cells and culture medium were added, and for the target cell maximum release well, 100 µL of target cells and 1% NP40 or 2.5% Triton were added. Three replicate wells were prepared for each condition and incubated in a 5% CO2 incubator at 37 °C for 4 h. After incubation, the 96‐well culture plate was centrifuged at 1500 rpm for 5 min. 100 µL of the supernatant was transferred to a flat‐bottom 96‐well culture plate, and 100 µL of LDH substrate solution was added for a 3‐minute incubation. Optical density (OD) was measured at 490 nm.

### Apoptosis Analysis

For flow cytometric analyses, cells subjected to different treatments were harvested, washed with PBS three times, and stained with the Annexin V‐FITC Apoptosis Detection Kit (Solarbio Inc.) according to the manufacturer's instructions 2 h after irradiation. The cells were detected using a flow cytometer.

### RNA Extraction and Real‐Time Quantitative qPCR to Check Gene Expression

The total amount of RNA extracted from the cell samples was determined using a TRIzol kit (Invitrogen Life Technologies). First‐strand cDNA was synthesized using the FastQuant RT Kit (TIANGEN, R6906) and qPCR was performed using SuperReal PreMix Plus (SYBR Green).

### Molecular Docking

The docking study was performed using Gide Docking Tool v. 7.9. The grid box was defined based on Walker B residues (296–315). The Van der Waals radii were set at 0.8 and the partial cutoff was 0.15. Ligand docking was performed in standard precision (SP) and extra precision (XP) modes with no constraints. Flexible ligand sampling, sampling nitrogen inversion, and ring conformation were used, adding bias sampling torsion penalization for amides with a nonplanar conformation and adding Epik state penalties to the docking score. To check for binding‐pose convergence, the top ten poses were included in the docking outputs. Poses and predicted hydrogen bonds were visualized using the PYMOL software.

### Proteome Analysis

B16, MLTCs and MDSCs cells were cultured in their respective suitable media at 37 °C with 5% CO₂ until the logarithmic growth phase. Then, the cells were collected by trypsin digestion, centrifuged, and the supernatant was discarded. Cell pellets were lysed on ice with pre – cooled lysis buffer (containing inhibitors) and then sonicated. After centrifugation, the supernatant was collected as the total protein. The protein concentration was measured with a quantification kit and adjusted to the same level. An appropriate amount of protein was taken and denatured in a boiling water bath with loading buffer to prepare samples. Liquid chromatography – tandem mass spectrometry (LC‐MS/MS) technology was used for analysis, and then The R Project was used for data processing to analyze the similarities and differences in the proteomes of the three cell types.

### mRNA Sequence Analysis

B16, MLTCs, and MDSCs cells were cultured under their respective appropriate conditions. After cell collection, total RNA was extracted. Once the quality inspection was qualified, the RNA was reverse – transcribed into cDNA, and a library was constructed. Then, quality detection and quantification were carried out for the library. After sample preparation, the samples were sent to Biomarker Technologies for mRNA sequencing analysis. Finally, BMKcloud tools were used to perform similarity and difference analysis on the sequencing data.

### Statistical Analysis

Statistical analyses were performed using GraphPad Prism 8, and the results were expressed as mean ± SD. Differences between groups were assessed through unpaired two‐tailed *t* test (for two‐sample comparison) or one‐way ANOVA with Dunnett's test (for multiple comparisons). Statistical significance was set at ns = not significant, *p < 0.05, **p < 0.01.

### Ethics Approval and Consent to Participate

All animal studies were carried out in accordance with the Animal Use Guidelines of National Institutes of Health and the current Chinese Regulations and Standards for the Use of Laboratory Animals. The experiment was approved in accordance with the guidelines of the animal ethics committee of Nankai University. Approval number: 2021‐SYDWLL‐000025.

## Conflict of Interest

The authors declare no conflict of interest.

## Author Contributions

T.S. and H.J.L. designed this study. T.S., H.J.L., Z.Y.L., and Y.N.L. write, review, and revise the manuscript. Z.Y.L., Z.Z.F., and M.Z.C. carried out the in vitro studies. Y.J.S. and Z.Y.L. participated in the animal experiments. XXS performed experiments and analyzed the results. Z.Z.F. and M.Z.C. performed part of the experiments. Z.Y.L., HZ, X.W.X and B.J.C. participated in the design of the study and performed the statistical analysis. Y.N.L., J.X.H., C.H.C., and Y.Q.F. conceived the study and participated in its design. All authors read and approved the final manuscript.

## Supporting information



Supporting Information

## Data Availability

The data that support the findings of this study are available from the corresponding author upon reasonable request.
