# Supplementary material for: Rbfox3 Promotes Transformation of MDSC‐Like Tumor Cells to Shape Immunosuppressive Microenvironment
Source: Adv Sci (Weinh). 2025 Jan 7;12(8):2404585. doi: 10.1002/advs.202404585 (PMC11848546; doi:10.1002/advs.202404585)
Supplement: Supplementary file 1 — Supporting Information [file ADVS-12-2404585-s001.docx]

**Rbfox3 Promotes Transformation of MDSC-like Tumor Cells to Shape Immunosuppressive Microenvironment**

*Zhiyang Li^1,2^, Zhuangzhuang Feng^1^, Mengzhan Chen^1^, Xinxiu Shi^1^, Bijia Cui^1^, Yujie Sun^1^, Heng Zhang^1,2^, Yinan Li^1^, Caihong Chen^1^, Yiqian Feng^1^, Jingxia Han^1^, Xuewu Xing^3,#^, Huijuan Liu^1,#^, Tao Sun^1,#^*

^1^State Key Laboratory of Medicinal Chemical Biology and College of Pharmacy, Nankai University, Tianjin, 300450, China.

^2^Tianjin Key Laboratory of Early Druggability Evaluation of Innovative Drugs, Tianjin International Joint Academy of Biomedicine, Tianjin, 300457, China.

^3^Department of Orthopedics, Tianjin First Central Hospital, Tianjin, 300190, China.

^#^Corresponding author Email: tao.sun@nankai.edu.cn (T.S.), huijuan.liu@nankai.edu.cn (HJ.L.), [xuewuxing@nankai.edu.cn](mailto:xuewuxing@nankai.edu.cn) (XW.X.).

Figure S1-S10

Figure S1

#
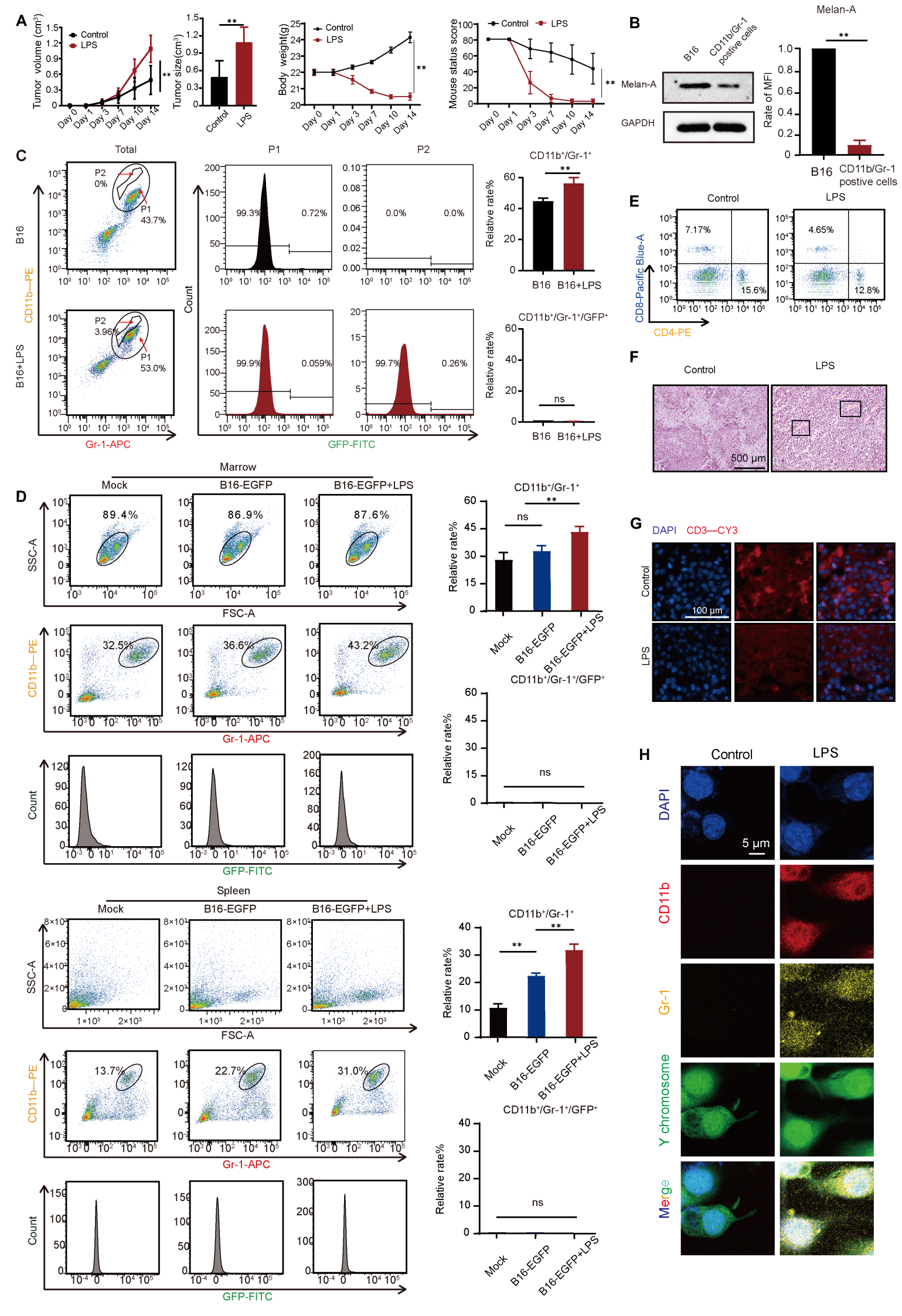


**Figure S1, related to Figure 1: Intratumoral injection of LPS into tumor-bearing mice in vivo induces B16 cell differentiation.** A) Statistical analysis of tumor size within 14 days of LPS injection into two groups of mice. Tumor volumes on day 14 were presented in the form of histograms and the changes were presented in the form of line graphs. Body weight and mouse status scores were presented as a line chart. B) Protein expression levels of PD-L1, and Tim-3 were detected by Western Blot analysis. Statistical analysis was performed, and the results were presented as histograms. C) Flow cytometry was performed to detect CD11b and Gr-1 signals in tumor cells isolated from B16 tumor-bearing mice or mice injected with LPS intratumorally, and CD11b/ Gr-1 double-positive cells were gated again to detect GFP signals. CD11b, Gr-1, and GFP-positive cells were quantified. D) Flow cytometry was used to detect the CD11b and Gr-1 signals in the spleen and bone marrow of mice in different groups, and CD11b/Gr-1 double-positive cells were gated to detect GFP signals. E) Flow cytometry of cytotoxic T cell content in tumor cells. F) Sections of tumor tissues from different groups. G) T cell fluorescence micrograph of tumor tissue sections. H) Immunofluorescence detection of male mouse-derived B16 subcutaneous tumor cells in female mouse tumor samples (CD11b, Gr-1, GFP, and DAPI). All values are presented as the mean ± SD, n = 6, ns = not significant, **p* < 0.05, ***p* < 0.01.

Figure S2

#
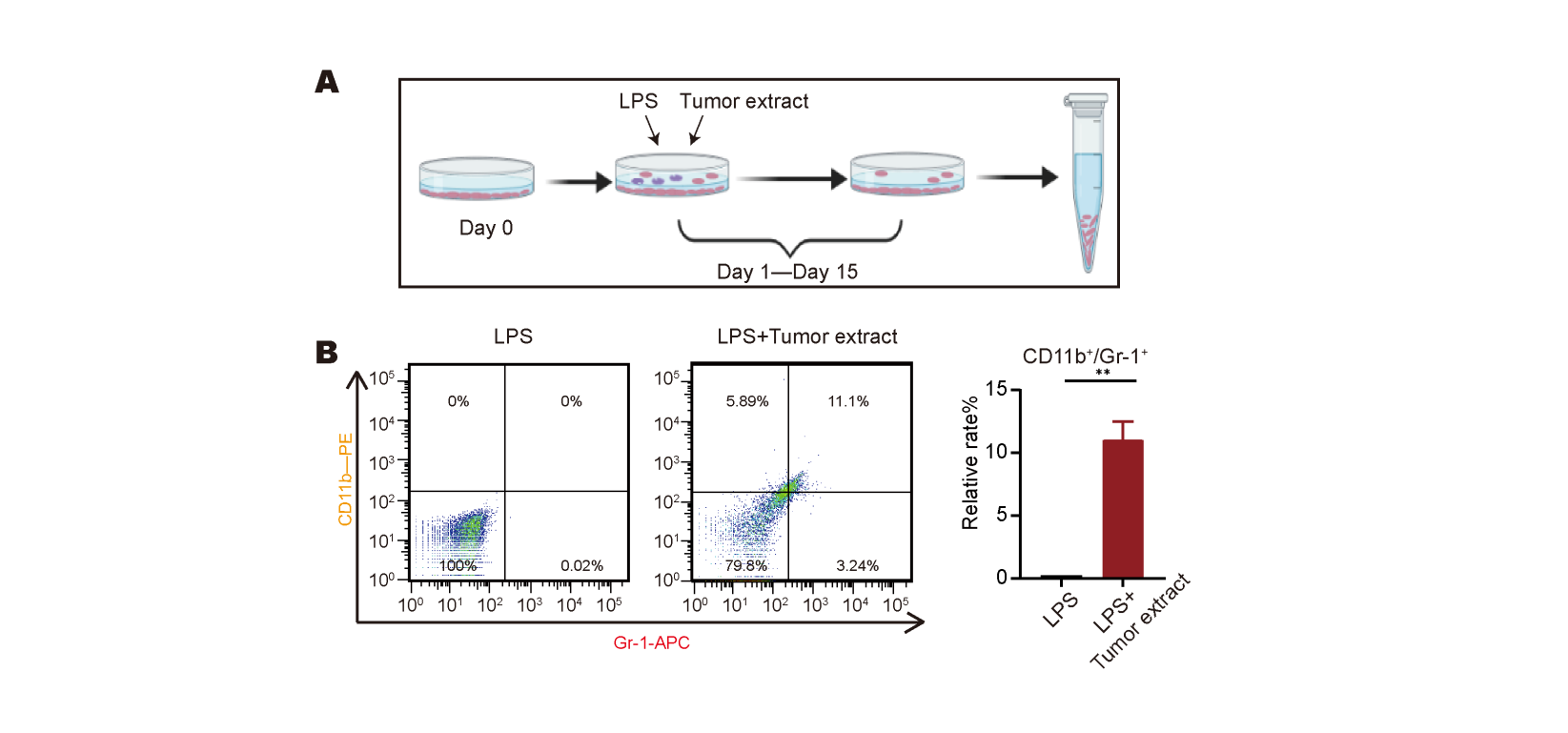
Figure S2, related to Figure 1: Under the induction of LPS and tumor extract, some B16 cells expressed CD11b and Gr-1 double-positive cells in vitro. A) Flow chart of culture conditions for the induction of B16 cell differentiation in vitro. B) Flow cytometry was used to detect CD11b and Gr-1 signals in LPS-induced group and LPS+ tumor extract-induced group, CD11b and Gr-1 positive cells were quantified. All values are presented as the mean ± SD, n = 6, ***p* < 0.01.

Figure S3

#
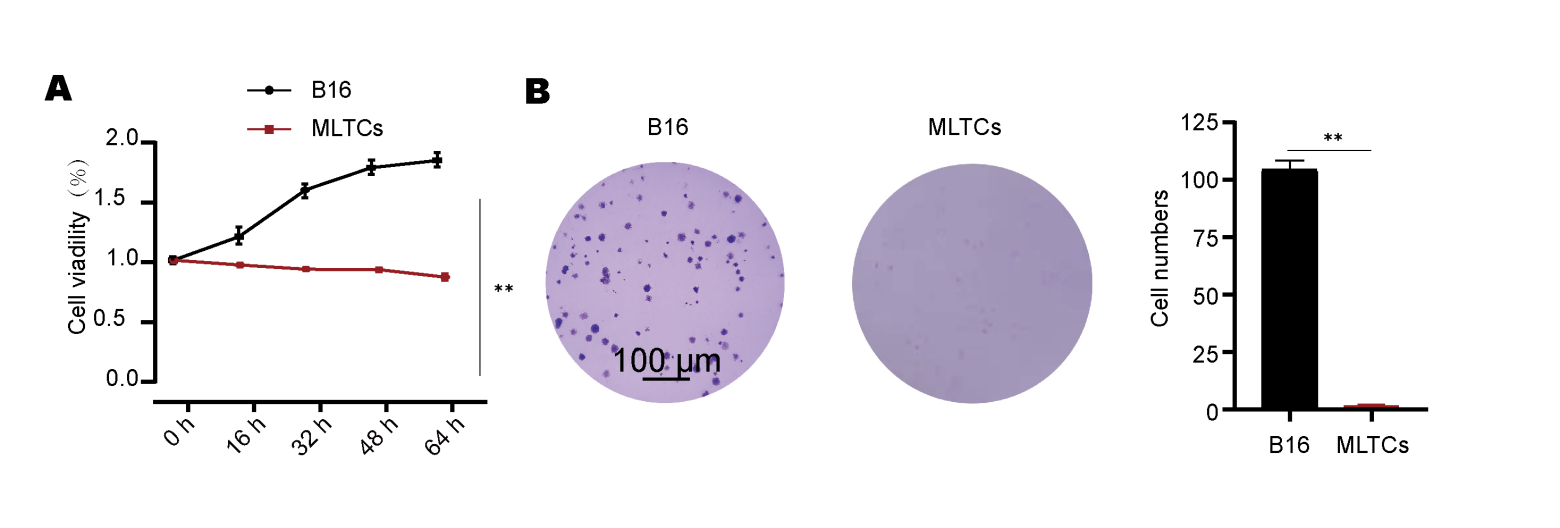


# Figure S3, related to Figure 1: MLTCs have short-term survival ability but fail to prolife rate and lack tumorigenic ability alone in vivo. A) CCK8 assay for B16 and MLTCs cellular viability. B) Representative images and analysis results of B16 and MLTCs clonogenic assays. All values are presented as the mean ± SD, n = 6, ***p* < 0.01.

Figure S4

#
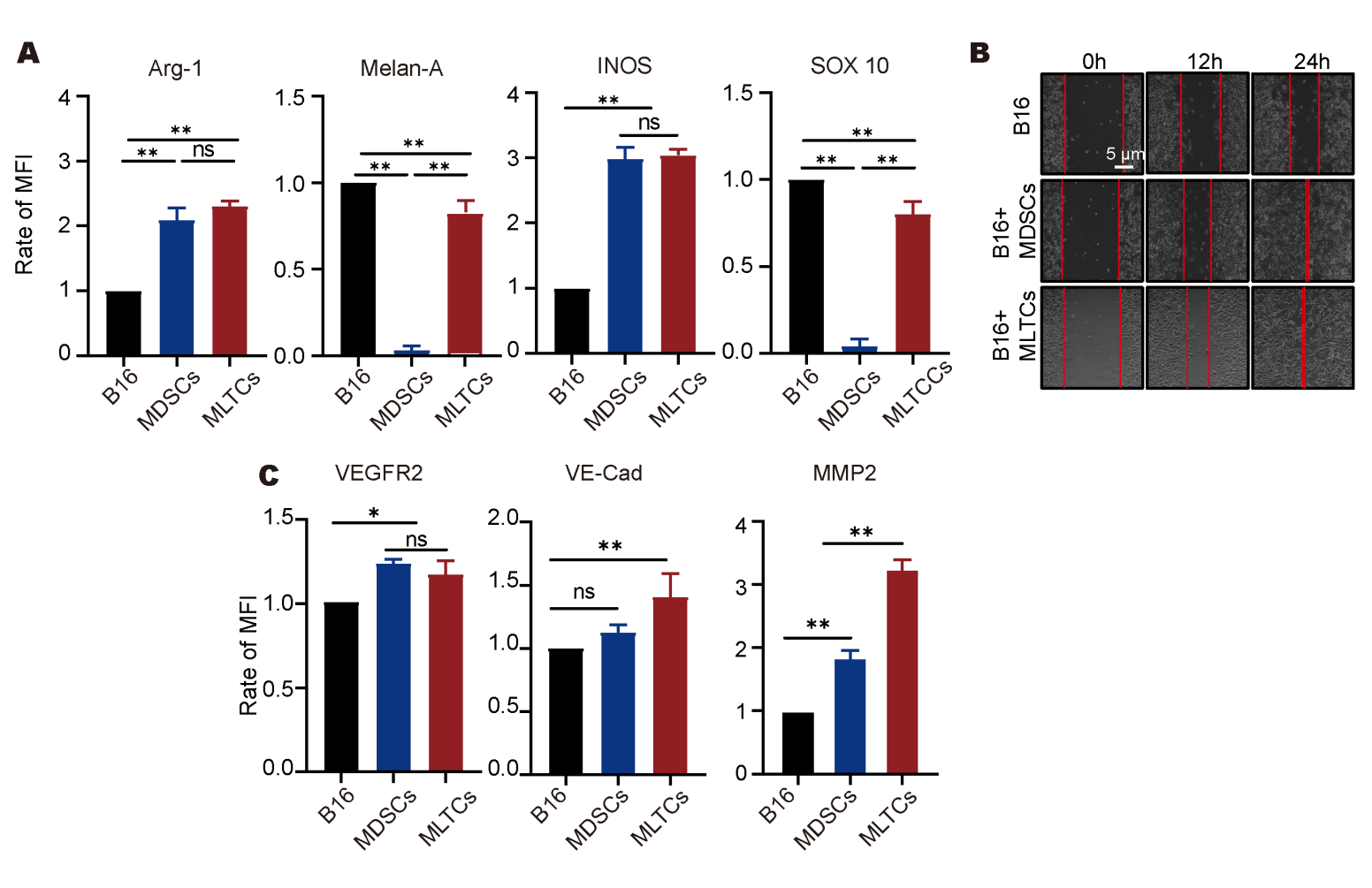


# Figure S4, related to Figure 2: MLTCs have the ability for immunosuppression and the promotion of the malignant evolution of tumor cells. A) Protein expression levels of iNOS and Arg-1 in B16 cells, MDSCs, and MLTCs were detected by Western Blot analysis. B) Representative plots of the scratch experiment at 0, 12, and 24 h. Red lines mark the edge of a scratch. C) Western Blot analysis was used to detect the changes in the expression levels of VEFGR2, VE-Cad and MMP2. Statistical analysis was performed, and the results were presented as histograms. All values are presented as the mean ± SD, n = 6, ns = not significant, **p* < 0.05, ***p* < 0.01.

Figure S5


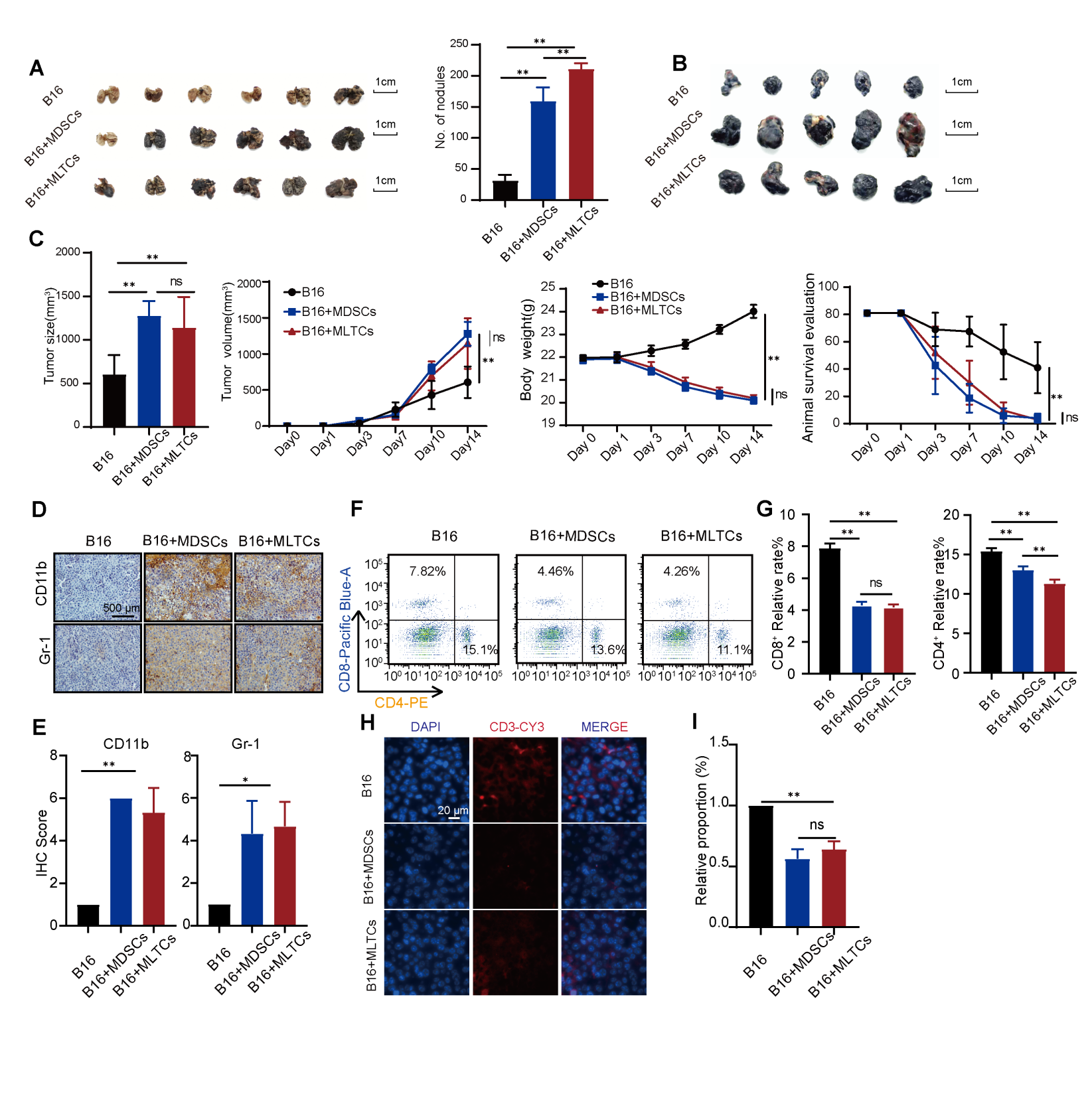


# Figure S5, related to Figure 2: MLTCs promote tumorigenesis and tumor development in vivo. A) Representative images of lung tumor metastasis in the three groups of tail vein-injected mice and histograms of the quantitative analysis of the number of lung metastatic focus points. B) Representative tumor images of the three groups of subcutaneously injected mice. C) Statistical analysis of tumor size in the three groups of mice. Results of the tumor volume were presented in the form of histograms, body weight and mouse status score were in the form of broken line graphs. D, E) Representative images and scoring of tumor-bearing mice immunostained for CD11b and Gr-1. Scoring criteria: positive rate (1–3 points), degree of positivity (1–3 points). Results were presented as a histogram. F, G) Flow cytometry analysis of cytotoxic T cell content in tumor cells. The results of CD8^+^ and CD4^+^ positive cells were presented as histograms. I, J) T cell immunofluorescence plots of tumor tissue sections of different treatment groups of tumor-bearing mice. Statistical analysis was performed, and relative proportions were presented in the form of histograms. All values are presented as the mean ± SD, n = 6, ns = not significant, **p* < 0.05, ***p* < 0.01.

Figure S6

**
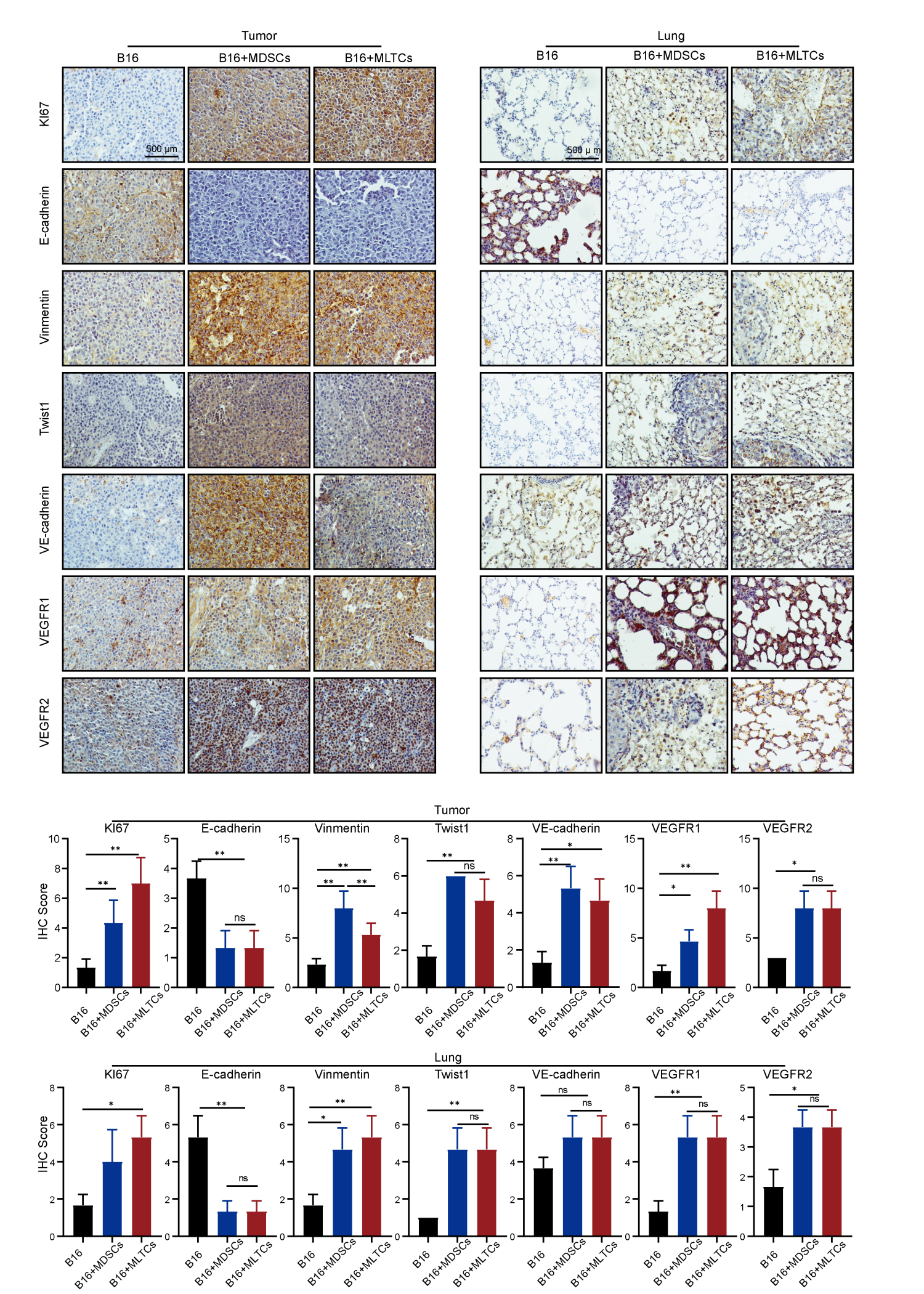
**

# Figure S6, related to Figure S5: Representative images of tumor immunohistochemistry. Representative immunohistochemical imaging and scoring of the tumor and lung tissues of tumor-bearing mice in different treatment groups. Scoring criteria: positive rate (1–3 points), degree of positivity (1–3 points). Results were presented as a histogram. All values are presented as the mean ± SD, n = 6, ns = not significant, **p* < 0.05, ***p* < 0.01.

Figure S7

#
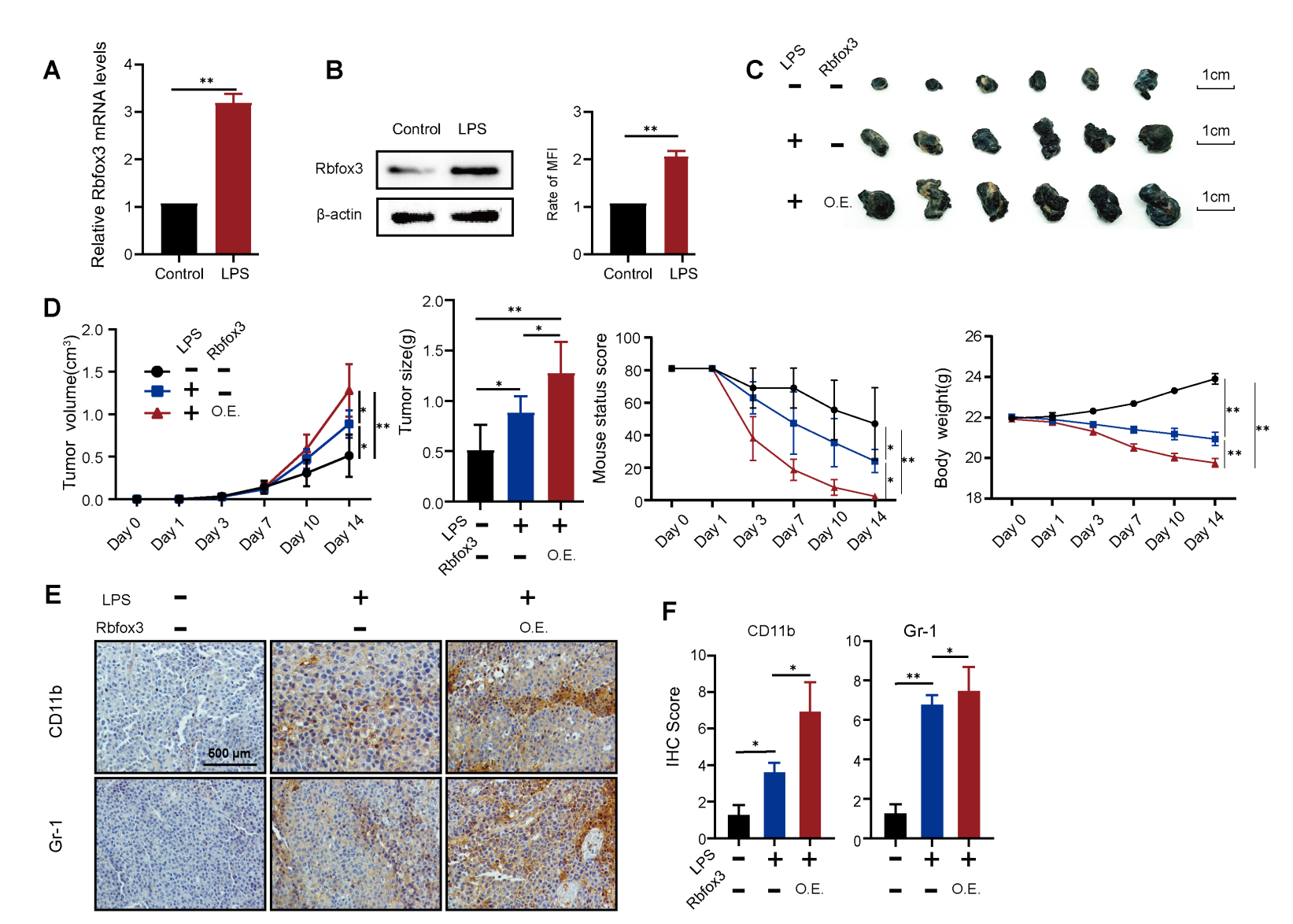
Figure S7, related to Figure 3: The quality of life of mice decreased after subcutaneous injection of B16 cells overexpressing the Rbfox3 gene. A, B) Rbfox3 mRNA levels and protein contents were determined by PCR and Western Blot analysis. The results of statistical analysis were presented as histograms. C, D) Representative images of tumors in tumor-bearing mice. Statistical analysis was performed on tumor size, and the results were presented in the form of histograms. The tumor volume, body weight and status score of mice were presented as line charts. E) Representative immunohistochemistry images of CD11b and Gr-1 in tumor-bearing mice were collected and scored. Scoring criteria: positive rate (1–3 points), degree of positivity (1–3 points). The results were presented as a histogram. All values were presented as the mean ± SD, n = 6, **p* < 0.05, ***p* < 0.01.

Figure S8

#
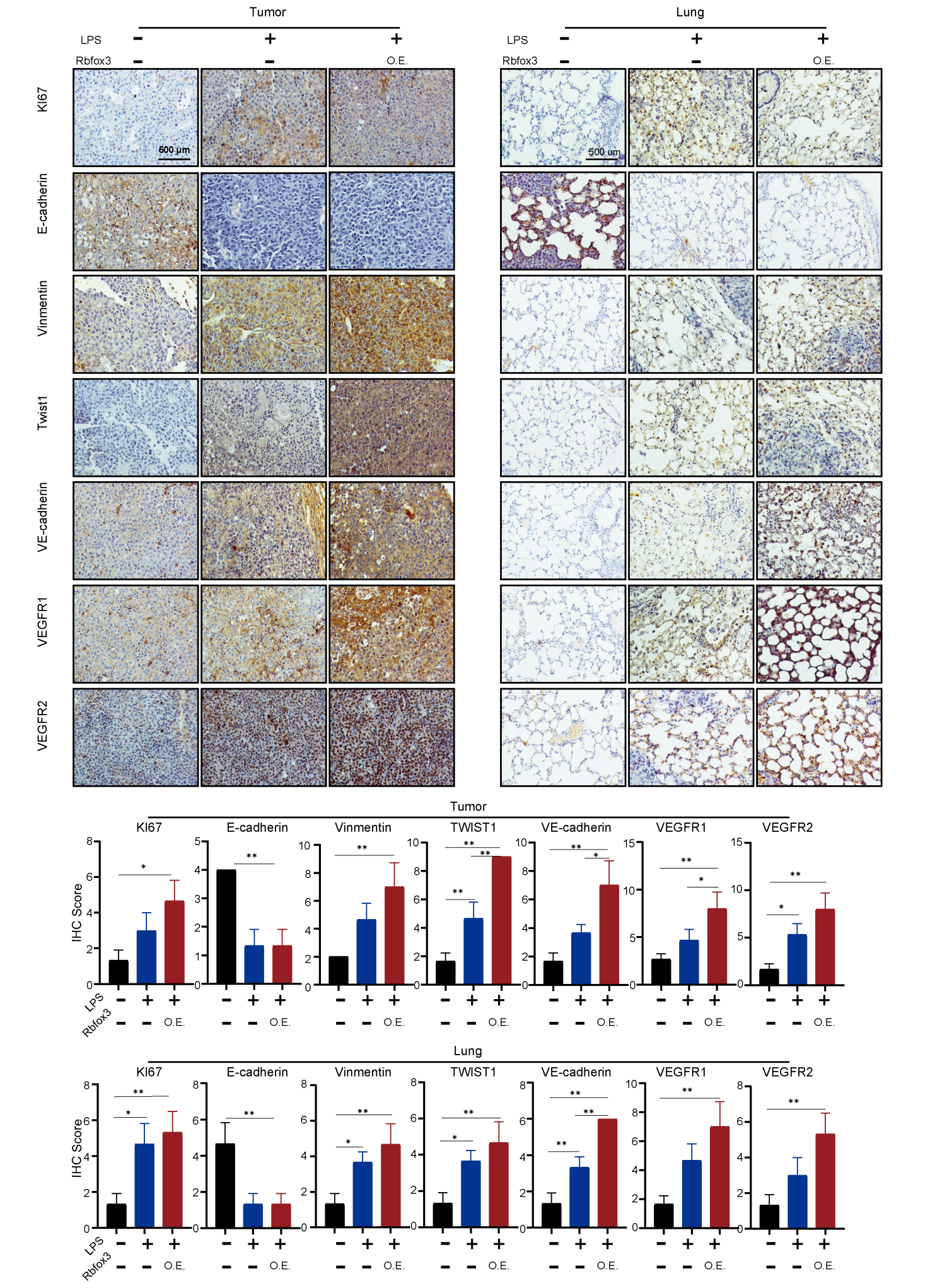


# Figure S8, related to Figure S7: Representative images of tumor immunohistochemistry. Representative immunohistochemical images and scores of the tumor and lung tissues of tumor-bearing mice in different treatment groups. Scoring criteria: positive rate (1–3 points), degree of positivity (1–3 points). The results were presented as a histogram. All values are presented as the mean ± SD, n = 6, **p* < 0.05, ***p* < 0.01.

Figure S9

#
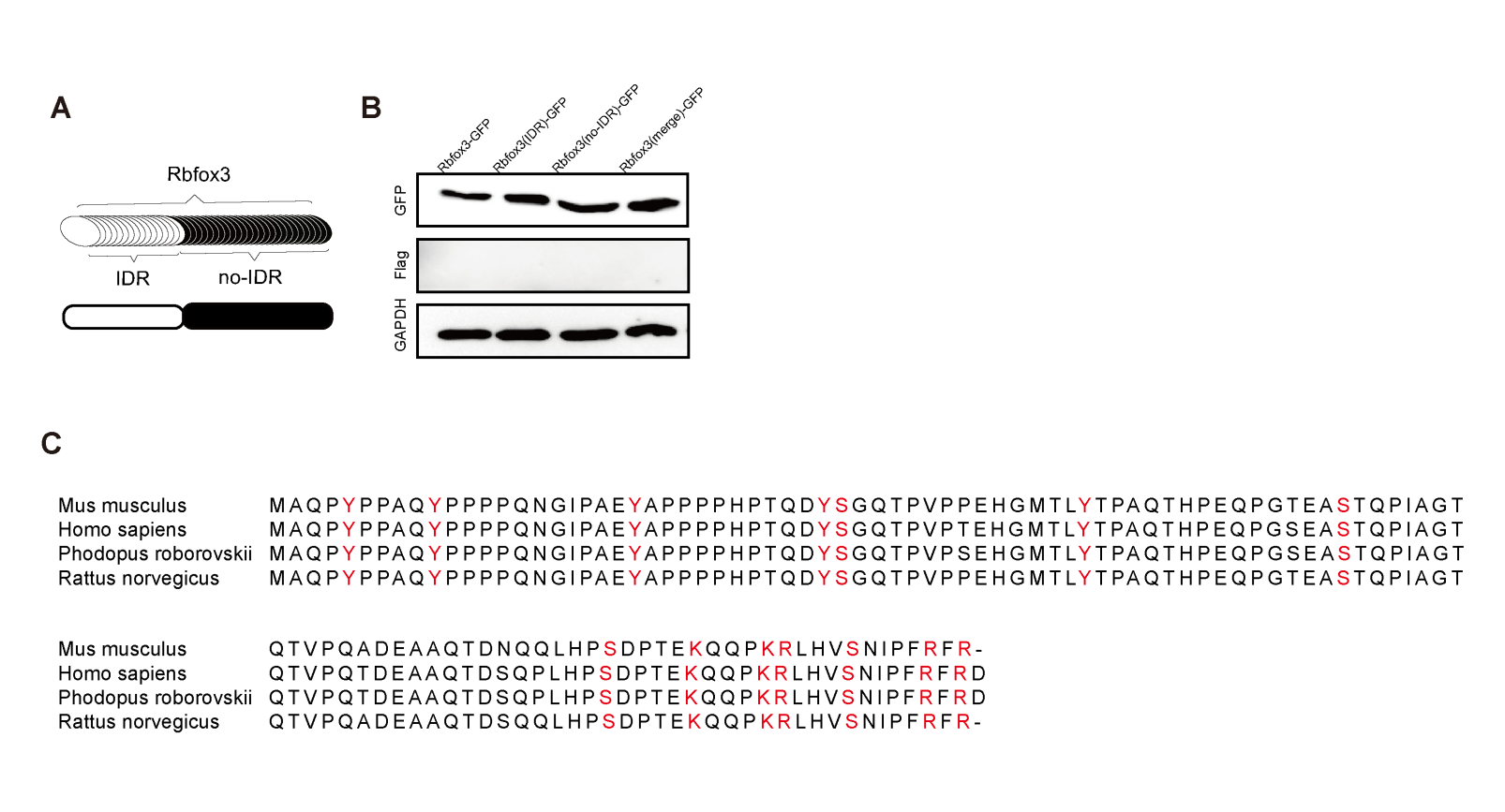


# Figure S9, related to Figure 5: Different protein designs based on Rbfox3. A) Rbfox3 proteins were classified into IDR and no-IDR. B) Western Blot analysis was performed to detect the levels of GFP and Flag proteins in four groups of proteins. C) Multiple sequence alignment of the Rbfox3 IDR in different species. The conserved K, R, S, and Y amino acid residues in IDRs were colored.

Figure S10


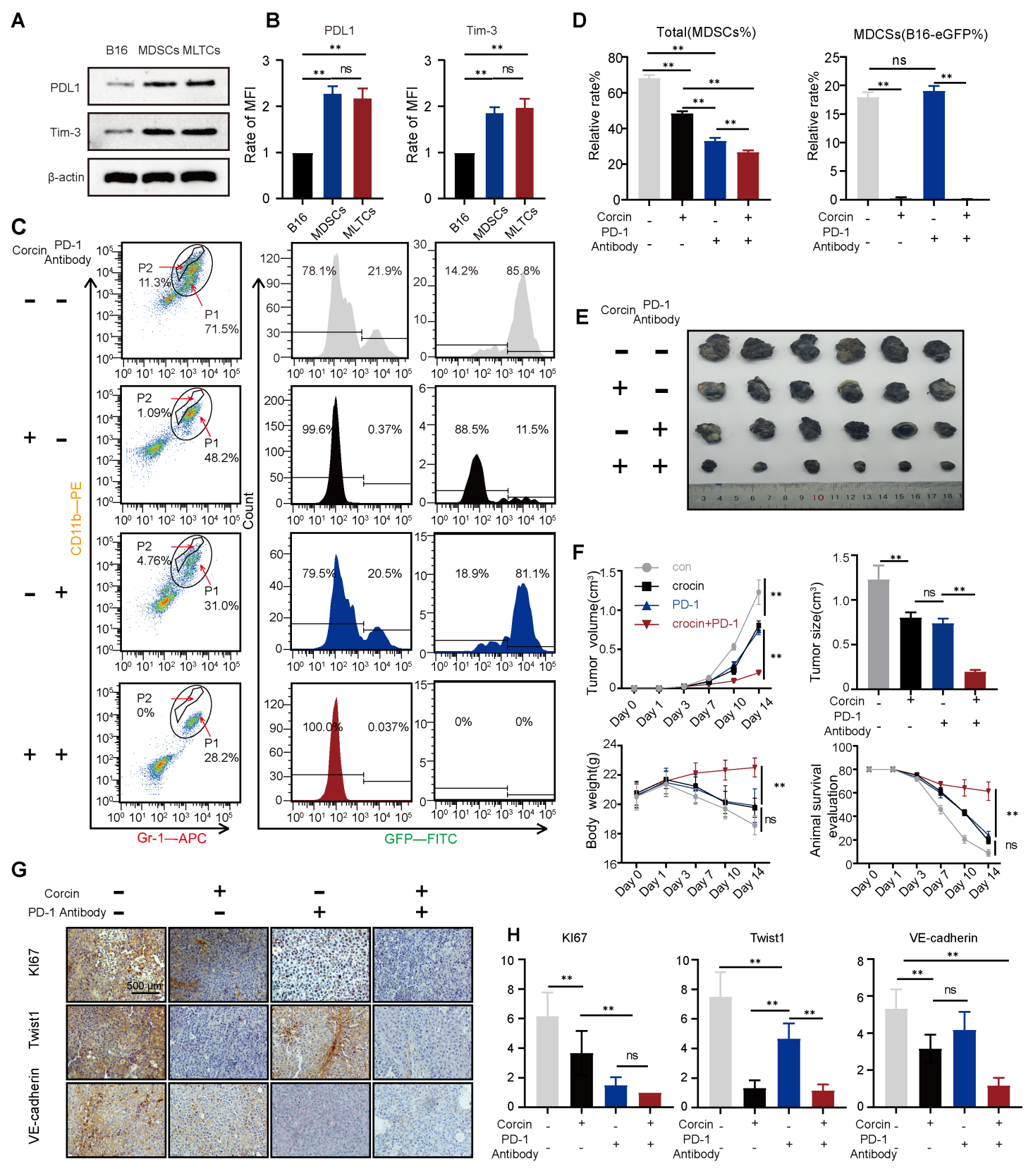


**Figure S10, related to Figure 6: Crocin effectively inhibits tumor growth and development in combination with the PD-1 antibody.** A-B) PD-L1, Tim-3 protein contents were determined by Western Blot analysis. C, D) Flow cytometry was performed to detect CD11b and Gr-1 signals in tumor-bearing mice, and CD11b/Gr-1 double-positive cells were gated again to detect GFP signals. E, F) Representative images of tumors in tumor-bearing mice. Tumor size was presented in the form of histograms, and tumor volume, mouse weight, and the mouse status score of mice were presented in the form of broken line graphs. G, H) Representative images and scoring of Ki67, Twist, and E-cadherin immunohistochemistry in tumor-bearing mice in different administration groups. Scoring criteria: positive rate (1–3 points), degree of positivity (1–3 points). Results were presented as a histogram. All values are presented as the mean ± SD, n = 6, ns = not significant, ***p* < 0.01.

**Primers**

Rbfox3: Forward: CACCACTCTCTTGTCCGTTTGC

Reverse: CACCACTCTCTTGTCCGTTTGC

Stxbp5: Forward: GGAGTGACAAAGGACAGCATCC

Reverse: TGCTGAGGAGGCTCACCATCTA

Ass1: Forward: CACTCTACGAGGACCGCTATCT

Reverse: CTCAAAGCGGACCTGGTCATTC

Gapdh: Forward:CATCACTGCCACCCAGAAGACTG

Reverse: ATGCCAGTGAGCTTCCCGTTCAG

**Plasmid**

All plasmid sequences were purchased from TSINGKE. Lentiviral knockout (K.O.) RNA was purchased from (Obio)

According to the manufacturer’s instructions, the cells were transfected with the Lipo8000 Transfection Reagent (Beyotime Biotechnology, China) into the cells for protein expression. After 48h, the cells were selected with G418 (1 mg/mL) for stable incorporation. Over-expression efficiency was determined by Western Blot analysis and RT-qPCR. The expression of genes was overexpressed or knocked down by using lentivirus. After 48h the cells were picked by resistance to puromycin.

**Primary antibody**

| **Name** | **Supplier** | **Catalogue number** |
| --- | --- | --- |
| CD11b | Invitrogen | 12-0112-81 |
| Gr-1 | Invitrogen | 17-5931-81 |
| Rbfox3 | Proteintech | 26975-1-AP |
| iNOS | Proteintech | 18985-1-AP |
| TLR4 | Proteintech | 66350-1-lg |
| NF-κB | Affinity | AF2006 |
| Arg-1 | Proteintech | 66129-1-AP |
| TNF-α | Affinity | AF7014 |
| MyD88 | Cell signalingtechnology | 4283s |
| β-actin | Affinity | AF7018 |
| Ki67 | Proteintech | 27309-1-AP |
| E-Cadherin | Cell signalingtechnology | 14472S |
| Vimentin | Affinity | AF7013 |
| Twist 1 | Affinity | AF4009 |
| VE-Cadherin | Immunoway | YT5611 |
| VEGFR1 | Affnity | AF6204 |
| VEGFR2 | Affnity | AF6281 |
| CD11b | Invitrogen | 14-0112-82 |
| Gr-1  CD33  HLA-DR | Invitrogen  Abcam  Abcam | 14-5931-82  ab269456  ab92511 |
| CD3  F4/80  CD133  SOX3  Oct3  Nanog  Anti-LPS | Bioss  Abcam  Abcam  Abcam  Abcam  Abcam  Abcam | Bsm-30092M  ab105080  ab252129  ab183606  ab183071  ab203919  ab35654 |

**Secondary antibody**

IF/ICC: Alexa Fluor 568-labeled Goat Anti-Mouse IgG antibodies, Alexa Fluor 647-labeled Goat Anti-Rabbit IgG antibodies, WB: Goat anti-rabbit IgG secondary antibody, Goat anti-mouse IgG secondary antibody (Beyotime), Immunohistochemistry: HRP-polymer anti-mouse/rabbit IHC Kit (Maixin Biotech, Fuzhou, China)
